# In vivo base editing by a single i.v. vector injection for treatment of hemoglobinopathies

**DOI:** 10.1172/jci.insight.162939

**Published:** 2022-10-10

**Authors:** Chang Li, Aphrodite Georgakopoulou, Gregory A. Newby, Kelcee A. Everette, Evangelos Nizamis, Kiriaki Paschoudi, Efthymia Vlachaki, Sucheol Gil, Anna K. Anderson, Theodore Koob, Lishan Huang, Hongjie Wang, Hans-Peter Kiem, David R. Liu, Evangelia Yannaki, André Lieber

**Affiliations:** 1 Department of Medicine, Division of Medical Genetics, University of Washington, Seattle, Washington, USA.; 2 Gene and Cell Therapy Center, Hematology Department, George Papanicolaou Hospital, Thessaloniki, Greece.; 3 Merkin Institute of Transformative Technologies in Healthcare, Broad Institute of MIT and Harvard, Cambridge, Massachusetts, USA.; 4 Department of Chemistry and Chemical Biology and; 5 Howard Hughes Medical Institute, Harvard University, Cambridge, Massachusetts, USA.; 6 Department of Computer Science and Biomedical Informatics, University of Thessaly, Lamia, Greece.; 7 School of Biology, Aristotle University of Thessaloniki, Thessaloniki, Greece.; 8 Hematological Laboratory, Second Department of Internal Medicine, Faculty of Health Sciences, School of Medicine, Aristotle University of Thessaloniki, Hippokration General Hospital, Thessaloniki, Greece.; 9 Stem and Gene Therapy Program, Fred Hutchinson Cancer Research Center, Seattle, Washington, USA.; 10 Department of Pathology, University of Washington, Seattle, Washington, USA.

**Keywords:** Hematology, Stem cells, Gene therapy, Hematopoietic stem cells, Monogenic diseases

## Abstract

Individuals with β-thalassemia or sickle cell disease and hereditary persistence of fetal hemoglobin (HPFH) possessing 30% fetal hemoglobin (HbF) appear to be symptom free. Here, we used a nonintegrating HDAd5/35++ vector expressing a highly efficient and accurate version of an adenine base editor (ABE8e) to install, in vivo*,* a –113 A>G HPFH mutation in the γ-globin promoters in healthy CD46/β-YAC mice carrying the human β-globin locus. Our in vivo hematopoietic stem cell (HSC) editing/selection strategy involves only s.c. and i.v. injections and does not require myeloablation and HSC transplantation. In vivo HSC base editing in CD46/β-YAC mice resulted in > 60% –113 A>G conversion, with 30% γ-globin of β-globin expressed in 70% of erythrocytes. Importantly, no off-target editing at sites predicted by CIRCLE-Seq or in silico was detected. Furthermore, no critical alterations in the transcriptome of in vivo edited mice were found by RNA-Seq. In vitro, in HSCs from β-thalassemia and patients with sickle cell disease, transduction with the base editor vector mediated efficient –113 A>G conversion and reactivation of γ-globin expression with subsequent phenotypic correction of erythroid cells. Because our in vivo base editing strategy is safe and technically simple, it has the potential for clinical application in developing countries where hemoglobinopathies are prevalent.

## Introduction

Autologous hematopoietic stem cell (HSC) gene therapy for hemoglobinopathies has shown promising clinical efficacy (1–4). However, current protocols involving isolation of patient HSCs, their in vitro genetic modification with integrating vectors, and reinfusion of the modified HSCs following myelotoxic BM conditioning are technically complex and expensive. We are attempting to develop an in vivo HSC gene therapy approach that does not require myeloablation and integrating vectors, and that is technically easier. In this approach, we are using capsid-modified, helper-dependent HDAd5/35++ vectors (1, 2). These vectors target CD46, a receptor that is expressed on primitive HSCs (2, 3). HDAd5/35++ vectors are injected i.v. after mobilization of HSCs from the BM by agents routinely used for HSC mobilization/harvesting. Mobilized HSCs are transduced while they are in the periphery. A large fraction of HSCs returns to the BM. Mobilization of HSCs is critical for in vivo transduction because, in the BM, they are surrounded by extracellular stroma proteins (4) and are not accessible to gene transfer vectors (2). To expand in vivo–transduced HSCs, we currently use an in vivo selection mechanism based on a mutant O^6^-methylguanine-DNA methyltransferase (*mgmt^P140K^*) gene that confers resistance to O^6^-BG/BCNU (O^6^-Benzylguanine/Carmustine) given at doses that are 20- to 30-fold lower than those used for cancer chemotherapy (5–7). We have demonstrated the safety (including the absence of clonal dominance; refs. 8–10) and efficacy of the in vivo approach in mice (9–15) and rhesus macaques (3, 8, 16). One of our goals is to broadly apply this approach for the treatment of hemoglobinopathies — i.e., β-thalassemia and sickle cell disease (SCD). Because of its simplicity and lower costs, this method could be applicable in developing countries where the burden from these diseases is high (17).

β-Thalassemia and SCD are the most common inherited diseases in humans worldwide (18). β-Thalassemia is caused by mutations in the *β-globin* gene that result in absent (β^0^/β^0^) or deficient (β^+^/β^+^, β^+^/β^0^) β-globin chain synthesis. Patients with β-thalassemia have a multitude of pathological symptoms and die prematurely (18). Most patients with SCD are homozygous for a Glu6Val substitution, resulting in the production of sickle β-globin (β^S^-globin). Clinically, SCD is characterized by recurrent episodes of severe bone pain, multiorgan failure, and early mortality. Individuals with hereditary persistence of fetal hemoglobin (HPFH) and thalassemic β-globin mutations exhibit few or no pathological effects because fetal hemoglobin (HbF) inhibits hemoglobin precipitation by reversing the globin chain imbalance and improving erythropoiesis (19).

This led to a number of HSC gene therapy approaches aimed toward the reactivation of fetal/γ-globin — e.g., targeting 2 transcriptional repressors, ZBTB7A and BCL11A, that participate in the developmental silencing of the 2 γ*-globin* genes (*HBG1/G*γ and *HBG2/A*γ) through binding to their respective promoter *cis*-regulatory elements (20). An approach based on RNA interference to silence *bcl11a* expression (20) or reactivation of γ-globin expression by CRISPR-Cas9 editing of the erythroid *bcl11a* enhancer (21) have delivered promising clinical data. Furthermore, preclinical studies to reactivate γ-globin by CRISPR/Cas9 editing of the BCL11A and ZBTB7A binding motifs within the *HBG1/2* promoters have shown efficient HbF reactivation (22). Along this line, we used an in vivo HSC transduction approach to express a CRISPR/Cas9 specific to the BCL11A binding site within the *HBG1/2* promoters and showed efficient γ-globin reactivation in transgenic mice carrying the WT 248 kb β-globin locus yeast artificial chromosome (β-YAC mice) (23). However, we also detected an undesired deletion of the *HBG1* gene due to CRISPR/Cas9-mediated double-stranded DNA breaks (DSBs) in both the *HBG1* and the *HBG2* promoter. There is also accumulating evidence that DSBs catalyzed by CRISPR/Cas9 can result in large genomic deletions and chromosomal rearrangements, as well as p53-induced cell cycle arrest and apoptosis (24–26). To address these problems, we focused our work on base editors, enzymes that are capable of introducing precise cytidine or adenine substitutions with minimal occurrence of DSBs and indels at the target site (27–30). Here, we employed an advanced adenine base editor version (ABE8e). ABE8e contains additional mutations that increase on-target activity and greatly decrease off-target activity (31).

**Figure 1:**
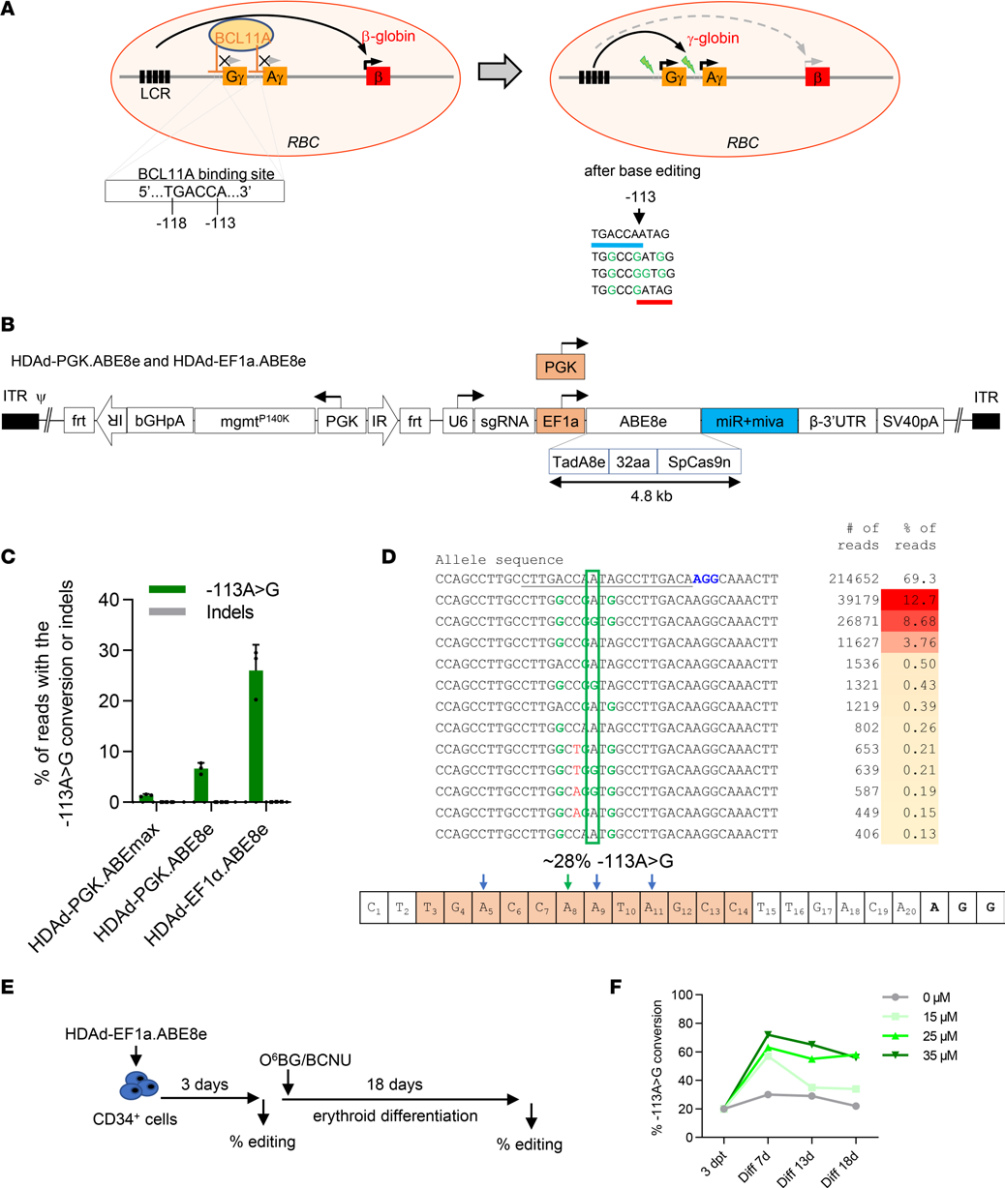
In vitro studies with HDAd-ABE8e vectors in CD34^+^ cells from healthy donors. (**A**) Reactivation of γ-globin by *HBG1/2* base editor. In adult erythroid cells, expression of γ-globin is inhibited by a number of repressor proteins, including BCL11A, which binds within the promoter of the 2 copies of the *HBG* gene (*HBG1*/Gγ and *HBG2*/Aγ). This directs the action of the strong β-globin locus control region (LCR) toward expression of β-globin. LCR activity can be switched back to the γ-globin by targeting the BCL11A binding motif using an adenine base editor (31). Expected outcomes of editing the –118 to –113 TGACCA BCL11A binding motif by ABE8e are shown on the bottom. In addition to the target –113 A>G conversion, bystander editing of neighboring adenines was observed. The –116 A>G conversion would further destroy the BCL11A binding motif (underlined blue). In the absence of the –110 A>G bystander conversion, a GATA motif would be created (underlined red). (**B**) The vectors contain an *HBG1/2* sgRNA and the *ABE8e* gene linked to miRNA regulatory elements. The *ABE8e* gene is either under the control of the PGK promoter (HDAd-PGK.ABE8e) or the EF1α promoter (HDAd-EF1α.ABE8e). The vectors also contain a PGK-mgmt^P140K^ cassette for O^6^-BG/BCNU selection. The *IR* and *frt* sequences are remnants from previous vectors that were integrated by SB100x transposase. They are irrelevant for this study, which does not employ the SB100x integrating system. (**C**) Editing rate in CD34^+^ cells from 3 healthy, G-CSF–mobilized donors. Cells were transduced at an MOI of 2,000 viral particles (vp)/cell, and 3 days later, genomic DNA was analyzed by Sanger sequencing for the –113 A>G conversion. ABEmax is an early adenine base editor version (12). (**D**) Sequences of the top 12 edited alleles in CD34^+^ cells from donor #1 after transduction with HDAd-EF1α.ABE8e. The target site –113 A (A8 in the sequence shown in the lower panel) is marked by a green box. Editable window in the spacer by ABE8e is highlighted in orange. (**E** and **F**) In vitro transduction studies with donor #1 CD34^+^ cells that were subjected to erythroid differentiation (ED) and 1 cycle of O^6^-BG/BCNU selection. (**E**) Schematic of the experiment. (**F**) Percentage of –113 A>G conversion at different time points without or with O^6^-BG + 15, 25, or 35 μM BCNU added at day 3. The SD was less than 10% for all time points. *n* = 3 technical replicates.

We targeted ABE8e to the BCL11A binding site in the *HBG1/2* promoters (Figure 1A) to mediate a A>G conversion at the –113 position. The corresponding sgRNA was selected in an earlier study from a series of sgRNAs targeting the BCL11A binding motif as the most efficient in γ-globin reactivation (12). It is thought that the –113 A>G mutation does not disrupt BCL11A binding, but rather creates a de novo binding site for the transcriptional activator GATA1 ([T/A]GATA[A/G]) that outcompetes binding of the BCL11A (32). However, as a result of the relatively wide editing window of ABE8e, in addition to the –113 A>G conversion, bystander editing at neighboring adenines is expected (Figure 1A, right panel). Specifically, the –116A>G conversion would destroy the BCL11A binding motif.

Here, we evaluated an HDAd-EF1α.ABE8e vector in vitro in CD34^+^ cells from patients with β-thalassemia and SCD, as well as in ex vivo and in vivo HSC gene therapy settings in the β-YAC mouse model. In this context, we tested a regimen of base editing that does not require the integration of the mgmt^P140K^ cassette for in vivo selection.

## Results

### HBG1/2 promoter editing in healthy CD34^+^ cells.

We used HDAd5/35++ vectors to express ABE8e either under the control of the phosphoglycerate kinase (PGK) promoter (HDAd-PGK.ABE8e) or the strong EF1α promoter (HDAd-EF1α.ABE8e) (Figure 1B). The HDAd vectors also contained a human mgmt^P140K^ expression cassette that allows for enrichment of edited cells after treatment with O^6^-BG/BCNU. To produce HDAd-EF1α.ABE8e, suppression of ABE8e expression on HDAd producer cells was required. This was achieved by placing the *ABE8e* gene under the regulation of miR–183-5p, miR–218-5p (33), and dual miR/virus-associated RNAI (miR-vaRNAI) (34). The first strategy makes use of 2 miRNAs, hsa-miR–183-5p and hsa-miR–218-5p, expressed exclusively by the HDAd producer cells. The second strategy makes use of a miRNA expressed by the helper virus called miR-vaRNAI. Because these miRNAs are only present during vector production, transgene expression from the HDAd is unimpeded in the transduced target. In addition, we further modified the helper vector by inserting expression cassettes for anti-CRISPR peptides capable of blocking ABE8e activity (Supplemental Figure 1; supplemental material available online with this article; https://doi.org/10.1172/jci.insight.162939DS1) (35). The editing rate of HDAd-ABE8e vectors was tested in human CD34^+^ cells from 2 healthy donors at day 3 after transduction (Figure 1C). HDAd-EF1α.ABE8e catalyzed, on average, approximately 26% of target site –113 A>G conversions, while HDAd-PGK.ABE8e and HDAd-ABEmax (used in an earlier study; ref. 12) conferred 6.6% and 1.3% editing, respectively. Subsequent studies, therefore, focused on HDAd-EF1α.ABE8e. Due to the wide editing window of ABE8e, in addition to the –113 adenine (shown as A8 at the bottom of Figure 1D), neighboring adenines (A5, A9, A11) can also be converted to guanines. To analyze the effect of editing on reactivation of γ-globin/HbF expression, HDAd-EF1α.ABE8e–transduced CD34^+^ were subjected to 18 days of erythroid differentiation (ED) with and without O^6^-BG/BCNU in vitro selection (Figure 1E). Target site conversion rates were measured at days 7, 13, and 18 of ED (Figure 1F). Starting at about 20% of editing, one-time treatment with of O^6^-BG plus 25 or 35 μM BCNU increased the editing rate to ~60% by day 18 of ED.

### HDAd-ABE8e HBG1/2 promoter editing, γ-globin reactivation, and phenotypic improvement in CD34^+^ cells from patients with β-thalassemia and SCD.

**Figure 2:**
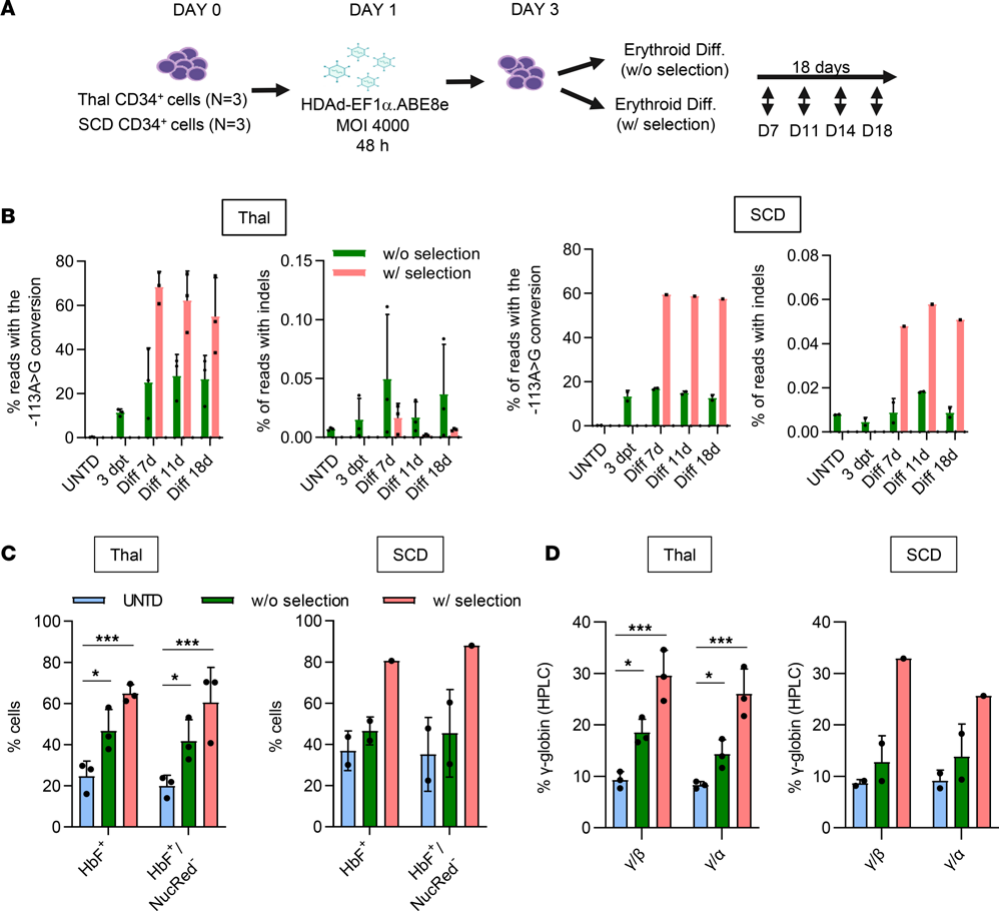
In vitro editing studies with CD34^+^ cells from patients with β-thalassemia and SCD. (**A**) Schematic of the experiment. G-CSF/plerixafor mobilized CD34^+^ cells from 3 β-thalassemia (Thal) patients were used. SCD CD34^+^ cells from fresh peripheral blood were isolated after blood transfusion of 3 patients (1 β^0^/β^S^, 2 β^S^/β^S^ patients). CD34^+^ cells were transduced with HDAd-EF1α.ABE8e or left untransduced (UNTD). Cells were then subjected to erythroid differentiation for 18 days. Aliquots were collected at the indicated time points. On day 3, 1 set of ED cells was treated once with O^6^-BG/BCNU for in vitro selection (only from second β^S^/β^S^ patient, we obtained sufficient numbers of CD34^+^ cell to perform in vitro selection). (**B**) Editing rate of the –113 A target site and percentage of reads with indels analyzed by NGS for the thalassemia samples (left panel) and SCD samples (right panel). Note the different scale on the *y* axes for on-target editing and indels. (**C**) Flow cytometry analysis for γ-globin/HbF at the end of ED in total cells and in differentiated enucleated erythroid (NucRed^–^) cells in Thal and SCD samples. (**D**) Measurement of globin protein chains by HPLC. Shown is the percentage of human γ-globin relative to human β- and α-globin chains in Thal samples (*n* = 3), 2 SCD (β^S^/β^S^) samples without selection, and 1 SCD (β^S^/β^S^) sample with selection. Statistical analyses of the data from the Thal samples were performed using 2-way ANOVA.

CD34^+^ cells were from 3 patients with β-thalassemia and 3 patients with SCD previously enrolled in mobilization trials. After transduction with HDAd-EF1α.ABE8e, cells were subjected to ED with and without in vitro selection (Figure 2A). Rates of –113 A>G conversion reached ~60% by next-generation sequencing (NGS) (Figure 2B) or by Sanger sequencing (Supplemental Figure 2) in HDAd-EF1α.ABE8e–transduced cells that were subjected to O^6^-BG/BCNU selection.

In ED cultures, the percentages of HbF^+^ cells in total erythroid cells and enucleated erythroid (NucRed^–^) cells were significantly higher in the HDAd-EF1α.ABE8e–transduced over the untransduced cells (Figs.2C, S3A). Notably, background γ-globin expression in the control vector–transduced samples is triggered by cytokines present in the culture medium (36). In vitro selection at day 3 further increased the percentage of γ-globin–expressing RBCs. Reactivation of γ-globin protein expression was also shown by HPLC, where γ-globin chains reached levels of 30% relative to β- and α-globin chains (Figure 2D).

**Figure 3:**
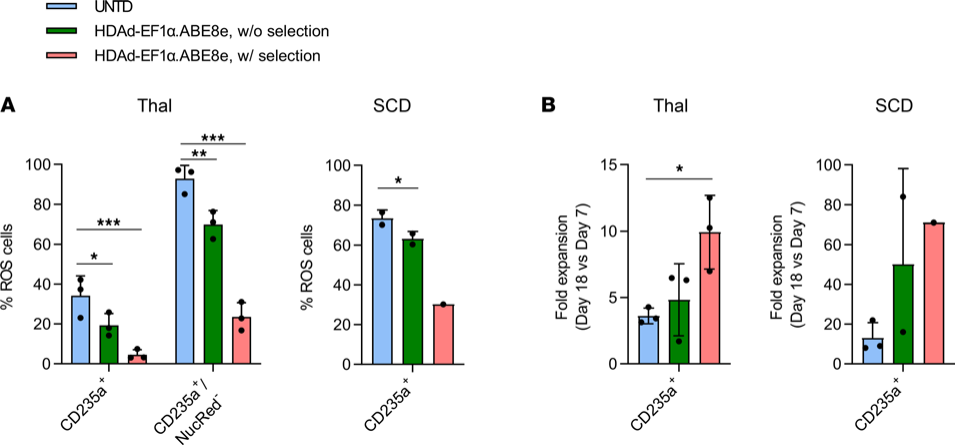
Phenotypic improvement in in vitro editing studies with CD34^+^ cells from patients with β-thalassemia and SCD. Analyses were done with the samples described in Figure 2. (**A**) Percentage of ROS^+^ cells within total erythroid cells (CD235a^+^) and denucleated erythroid cells (CD235a^+^/NucRed^–^ cells) for Thal samples and in total erythroid cells for SCD samples. (**B**) Fold increase in erythroid cell numbers between days 7 and 18 of ED. Statistical analyses of the data from the Thal samples were performed using 2-way ANOVA.

**Figure 4:**
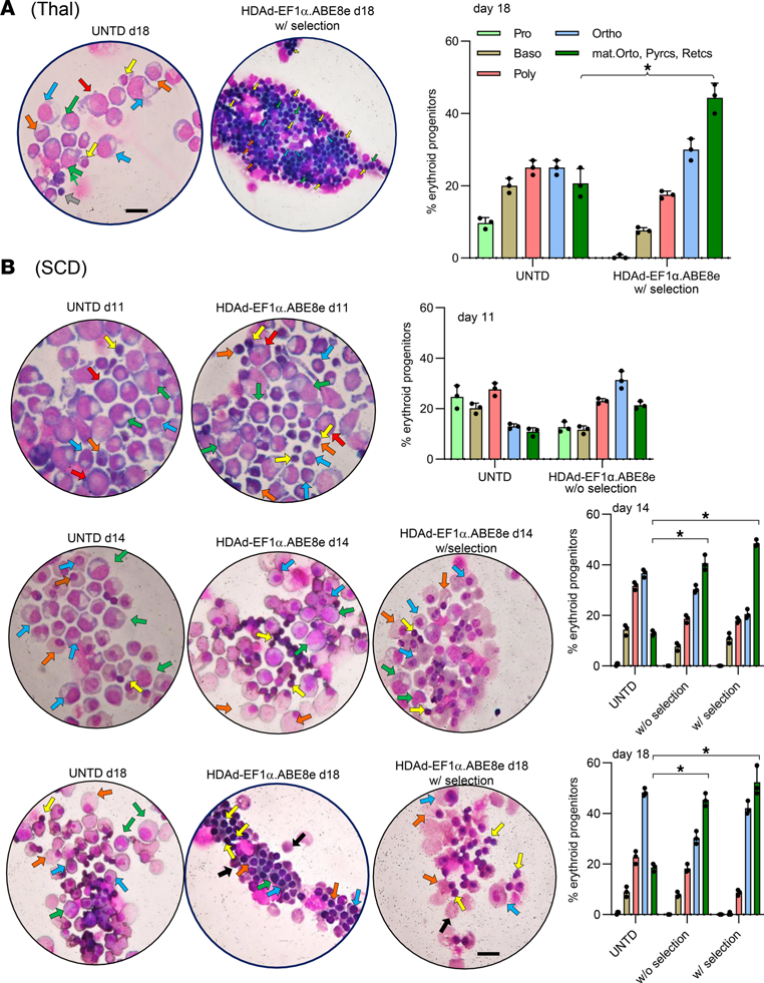
Cytospins from cultures at different time points of ED. (**A**) Representative samples from Thal CD34^+^ cells either untransduced or transduced with HDAd-EF1α.ABE8e and O^6^-BG/BCNU selected. Left panel: cytospins stained with Giemsa/May-Grünwald (see Supplemental Methods). The arrows indicate cells at different stages of erythroid differentiation: proerythroblasts (red) → basophilic erythroblasts (green) → polychromatic erythroblasts (blue) → orthochromatic erythroblasts (orange) → maturing orthochromatic erythroblasts (yellow) → reticulocytes (black)/pyrenocytes (gray). Scale bar: 25 μm. Right panel: quantification of erythroid progenitors. Five random fields were counted by 2 scientists. Pro, proerythroblasts; Baso, basophilic erythroblasts; Poly, polychromatic erythroblasts; Ortho, orthochromatic erythroblasts; mat; Ortho, maturing orthochromatic erythroblasts; Retics, reticulocytes; Pyrcs, pyrenocytes. (**B**) Cytospins from differentiated SCD CD34^+^ cells at days 11, 14, and 18 of ED, untransduced and HDAd-EF1α.ABE8e –transduced without and with selection. Statistical analyses were performed using 2-way ANOVA. *P < 0.05.

Efficient γ-globin reactivation resulted in a significant reduction of reactive oxygen species (ROS) levels, a hallmark of oxidative stress seen in β-thalassemia and SCD (37) (Figure 3A and Supplemental Figure 3B). γ-Globin reactivation by HDAd-EF1α.ABE8e also improved ED/expansion of β-thalassemia and SCD CD34^+^ cells. The total number of erythroid cells increased 2- and 7-fold, respectively, for β-thalassemia and SCD samples between days 7 and 18 in settings with in vitro selection (Figure 3B). Improved erythropoiesis is also supported by microscopic analyses of cells at the end of ED, and this analysis shows more differentiated cells (maturing orthochromatic erythroblasts, reticulocytes/pyrenocytes) in the β-thalassemia (Figure 4A) and SCD (Figure 4B) samples.

HDAd-EF1α.ABE8e transduction and base editing did not cause cytotoxicity as demonstrated by (a) no reduction in the percentage of primitive HSCs (CD34^+^CD38^–^CD90^+^) (Supplemental Figure 4A) and (b) no significant effect on progenitor colony formation (Supplemental Figure 4B).

In summary, transduction of CD34^+^ cells from patients with β-thalassemia and SCD with HDAd-EF1α.ABE8e mediated efficient editing of the *HBG1/2* target site and the reactivation of γ-globin expression, resulting in phenotypic amelioration of erythroid cells after in vitro ED of HSCs.

### Ex vivo HSC genome editing without O^6^-BG/BCNU selection.

**Figure 5:**
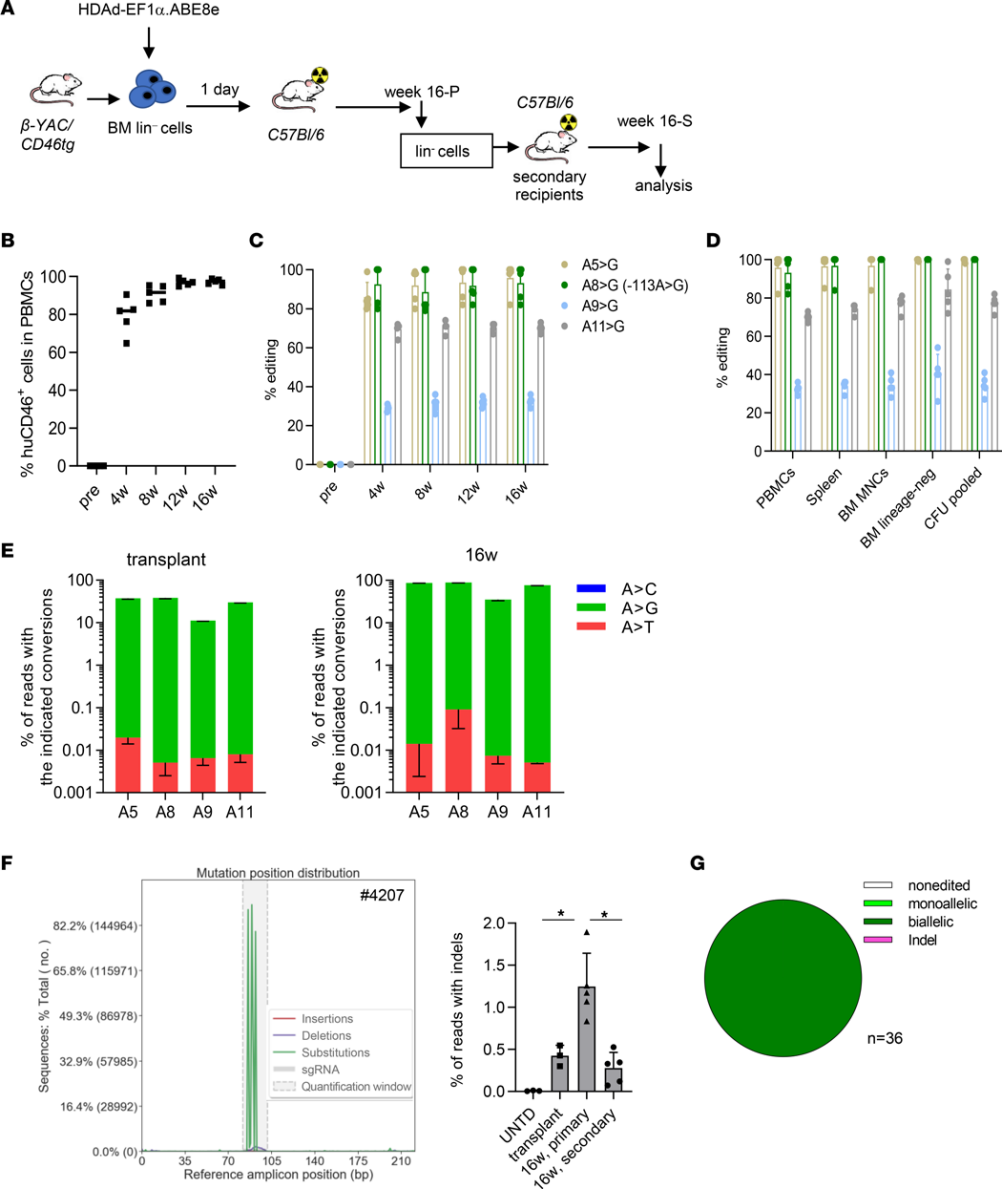
Ex vivo HSC base editing for γ-globin reactivation by HDAd-EF1α.ABE8e. (**A**) Schematic of the experiment. BM Lin^–^ cells were isolated from β-YAC/CD46 transgenic mice and transduced ex vivo with HDAd-EF1α.ABE8e at an MOI of 500 vp/cell. After 1 day in culture, 1 million cells per mouse were transplanted into lethally irradiated C57BL/6 mice, which were followed for 16 weeks (week 16 primary [week 16-P]). Data from these mice are shown in this figure. BM Lin^–^ cells from these mice were then used for secondary transplantation and these mice were monitored for another 16 weeks (week 16 secondary [week 16-S]; see Supplemental Figure 6). (**B**) Engraftment of transplanted HDAd-EF1α.ABE8e–transduced HSCs measured by flow cytometry of human CD46 in PBMCs. Each symbol is an individual mouse. *n* = 5 animals. (**C**) Analysis of target site editing in PBMCs by Sanger sequencing. Shown are percentages of conversion for the –113 A>G site and neighboring adenines. *n* = 5 animals. (**D**) Analysis of target site editing in PBMCs, spleen, BM MNCs, BM Lin^–^ cells, and CFU at week 16 after transplantation by Sanger sequencing. *n =* 5 animals. (**E**) Comparison of editing rates (by NGS) at the 4 adenines in the transplant (ex vivo transduced Lin^–^ cells cultured for 3 days) and BM MNCs at week 16 after transplantation. Shown are percentages of reads. Note the log_10_ scale of the *y* axis. *n* = 3 animals. (**F**) NGS of the target area (222 bp amplicon; ~100 nucleotides upstream and downstream of the spacer). Left panel: base substitutions (green), deletions (blue), and insertions (red) in the target area for 1 representative mouse. Right panel: summary of all indel reads in the transplant, week 16-P mice and week 16-S mice. *P < 0.05. Statistical analyses were performed using 2-way ANOVA. (**G**) Editing on a single cell basis. Week 16 BM Lin^–^ cells were plated for progenitor assay, and individual colonies were subjected to NGS. Shown is a representative mouse with 100% biallelic editing of the *HBG1/2* sites. *n* = 36 (3 mice, 12 colonies per mouse analyzed).

We used transgenic β-YAC mice including the locus control region (LCR), *γ-globin*, and *β-globin* genes (23). They were crossed with human CD46 transgenic mice to allow for HDAd5/35++ transduction via CD46. First, we tested an ex vivo HDAd-EF1α.ABE8e editing approach. BM lineage^–^ (Lin^–^) cells (a fraction enriched for HSCs) from β-YAC/CD46 mice were transduced with HDAd-EF1α.ABE8e and transplanted into lethally irradiated C57BL/6 mice (Figure 5A). Engraftment, measured based on human CD46 expression on PBMCs, was > 95% after week 8, indicating that ex vivo HSC transduction and editing did not affect HSC functions (Figure 5B). After transplantation, A>G conversion rates measured in PBMCs by Sanger sequencing were > 90% at the first time point analyzed (4 weeks after transplantation) and remained stable over the 16-week (week 16 primary [week 16-P]; see Figure 5A) observation period (Figure 5C). As seen in in vitro studies with human CD34^+^ cells (Figure 1D), substantial bystander A>G editing was observed, specifically at position A5. A>G conversion rates in splenocytes, BM mononuclear cells (MNCs), and Lin^–^ cells, as well as CFUs were in agreement with the PBMC data (Figure 5D). The comparison of editing rates in the transplanted cells (~38% –113 A>G) and in week-16-P mice (>95% –113 A>G) likely indicates preferential transduction of repopulating HSCs and expansion of edited cells after transplantation (Figure 5E). The vast majority of edits were A>G conversions (Figure 5E). The rate of A>T and A>C conversions was ~3 and ~4 orders of magnitude lower, respectively. NGS of the target area confirmed the A>G conversion rates detected by Sanger sequencing and determined that indels (mostly deletions) were < 1.5% of all reads (Figure 5F and Supplemental Figure 5). HSCs contain 2 *HBG* promoters (*HBG1* and *HBG2*) on each allele. To assess editing of the 4 target sites on a single-cell level, we isolated individual progenitor colonies and sequenced their DNA (*n* = 20 colonies). The data show 100% biallelic editing of both promoters (Figure 5G).

**Figure 6:**
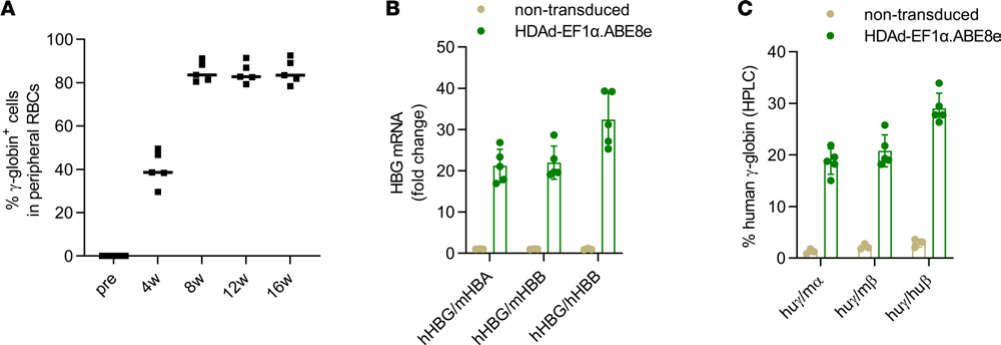
γ-Globin analysis after ex vivo base editing with by HDAd-EF1α.ABE8e. (**A**) Percentage of γ-globin^+^ peripheral RBCs measured by flow cytometry at different time points after transplantation. *n* = 5 animals. (**B**) qPCR for globin mRNA (human HBG/γ-globin versus mouse HBA/α-globin; human HBG/γ-globin versus mouse HBB/β-globin; human HBG/γ-globin versus human HBB/β-globin). Shown are the fold changes between untreated and treated animals. *n* = 5 animals. (**C**) Percentage of human γ-globin chains relative to mouse α-globin chains, mouse β-globin, and human β-globin chain measured by HPLC.

γ-Globin protein was detected by flow cytometry in 80%–90% of peripheral RBCs starting after week 8 (Figure 6A), in accordance with the HSC engraftment/expansion kinetics (Figure 5B) and previous studies with CRISPR/Cas9 (37). Relative γ-globin expression levels were measured on the mRNA and protein levels. *HBG* mRNA levels were approximately 35% of human *HBB* mRNA levels and approximately 22% of mouse *HBA* and *HBB* mRNA (Figure 6B). In mice transplanted with ex vivo–edited Lin^–^ cells, human γ-globin levels were about 30% of human β-globin chains and approximately 20% of mouse α- or β-globin chains (Figure 6C). Editing rates and γ-globin reactivation were stable in secondary recipients, demonstrating that editing occurred in long-term repopulating HSCs (Supplemental Figure 6).

In summary, HDAd-EF1α.ABE8e confers efficient ex vivo HSC editing without toxicity associated with the transduction and editing processes. After editing, γ-globin levels were 35% of human β-globin levels without any O^6^-BG/BCNU in vivo selection.

### In vivo HSC genome editing.

**Figure 7:**
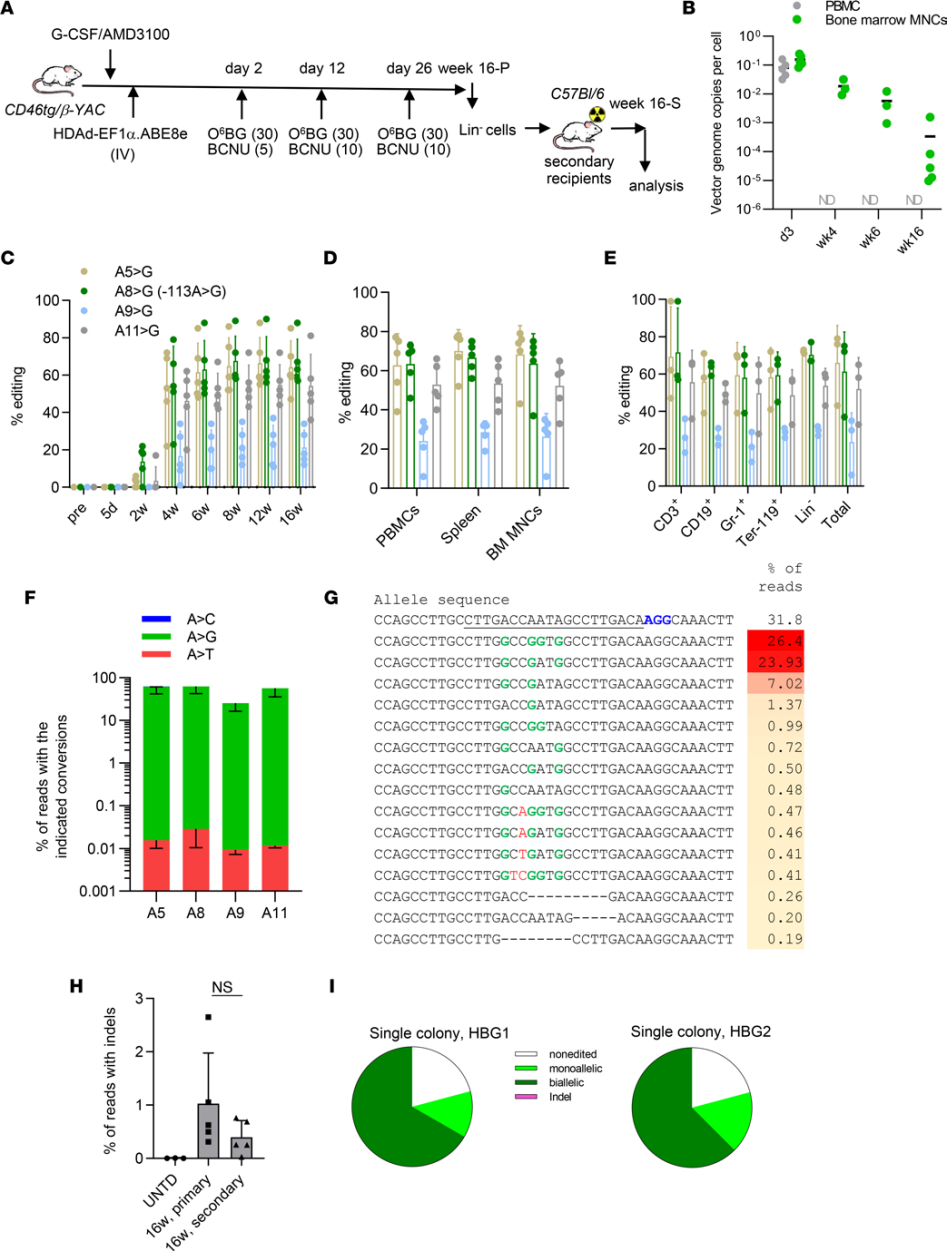
In vivo HSC transduction of β-YAC/CD46 mice to achieve γ-globin reactivation by HDAd-EF1α.ABE8e. (**A**) Experimental procedure. β-YAC/CD46 mice (*n* = 5 animals) were mobilized by G-CSF/AMD3100 and in vivo transduced by i.v. injection of HDAd-EF1α.ABE8e. In vivo selection with O^6^-BG/BCNU was started at day 2 after HDAd injection and repeated at days 12 and 26 at the indicated doses (30 mg/kg O^6^-BG + 5, 10, and 10 mg/kg BCNU). The mice were euthanized at week 16-P. The data from primary in vivo transduced mice are sown in this figure. Lin^–^ cells were isolated from BM and i.v. injected into lethally irradiated C57BL/6J mice. The secondary transplanted mice were followed for another 16 weeks (week 16-S; see Supplemental Figure 10). (**B**) Loss of vector genomes in PBMCs and BM MNCs. Vector copy number (VCN) was measured by qPCR with human mgmt^P140K^ primers. ND, not detectable. *n* = 5 animals for d3 and wk16; *n* = 3 animals for wk4 and wk6. (**C**–**E**) Target base conversion measured by Sanger sequencing. Each dot represents 1 animal. *n* = 5 animals. (**C**) Percent conversion in DNA from PBMCs. (**D**) Percent conversion at week 16 in PBMCs and MNCs in the spleen and BM. (**E**) Percent conversion at week 16 in lineage (CD3^+^, CD19^+^, Gr-1^+^, Ter-119^+^) cells and in Lin^–^ cells in the BM. (**F**) Comparison of editing rates at the 4 adenines in BM MNCs at week 16 after in vivo transduction. Shown are percentages of reads. *n* = 5 animals. (**G**) Representative NGS results showing target base conversion and indels at week 16 (mouse #446). (**H**) Summary of all indel reads in week 16-P mice and week 16-S mice. *n* = 5 animals. (**I**) Editing on a single-cell basis. Week 16 BM Lin^–^ cells were plated for progenitor assays, and individual colonies were subjected to NGS. Shown are sequencing data of the *HBG1* and *HBG2* sites of a representative colony with mono- and biallelic conversions, as well as indels (<1%, not visible).

One of our hypotheses was that, after in vivo HSC transduction, HDAd genomes and, consequently, ABE8e and mgmt^P140K^ expression would be gradually lost due to cell division and vector genome degradation (Supplemental Figure 7). We, therefore, designed a new in vivo HSC transduction/selection regimen shown in Figure 7A. After mobilization of CD46/β-YAC transgenic mice and i.v. injection of a single HDAd-EF1α.ABE8e vector, O^6^-BG/BCNU treatment was started at day 2 and repeated at days 12 and 26 to capitalize on mgmt^P140K^ expression from episomal HDAd-EF1α.ABE8e genomes and to dilute out the vector by stimulating cell division. The latter was demonstrated by qPCR that measured vector copy numbers (VCN) in BM MNCs. In in vivo transduced primary mice, during the 16-week experiment, the VCN declined by 2–4 orders of magnitude (Figure 7B), resulting in undetectable *ABE8e* and *mgmt^P140K^* mRNA levels at week 16 after transduction (data not shown). Base editing was measured by Sanger sequencing in PBMCs (Figure 7C). A>G conversion rates increased after selection to 50%–90% (average 67%) for the –113 target site with a similar pattern of bystander adenine editing seen in the ex vivo study. Furthermore, conversion rates were similar in PBMCs, splenocytes, and BM MNCs (Figure 7D). Editing rates were comparable for CD3^+^ (T cell), CD19^+^ (B cell), Gr-1^+^ (myeloid), and Ter-119^+^ (erythroid) lineages in the BM, indicating that editing occurred in multipotential HSCs and that *HBG1/2* editing (and γ-globin reactivation) did not provide a proliferative advantage or disadvantage for any specific lineage in β-YAC mice (Figure 7E). NGS demonstrated again a high specificity for A>G conversions with less than 1.5% of indels (Figure 7, F–H, and Supplemental Figure 8). Editing of the 4 *HBG* target sites on a single-cell level was less efficient than in the ex vivo setting (Figure 7I). In total, 66.7% and 71.4% of colonies had biallelic conversion in the *HBG1* and *HBG2* promoters, respectively. A total of 21% (*HBG1*) and 23.6% (*HBG2*) of colonies had monoallelic –113 A>G conversions. The percentages without *HBG1* and *HBG2* editing were 21% and 23.8%, respectively.

**Figure 8:**
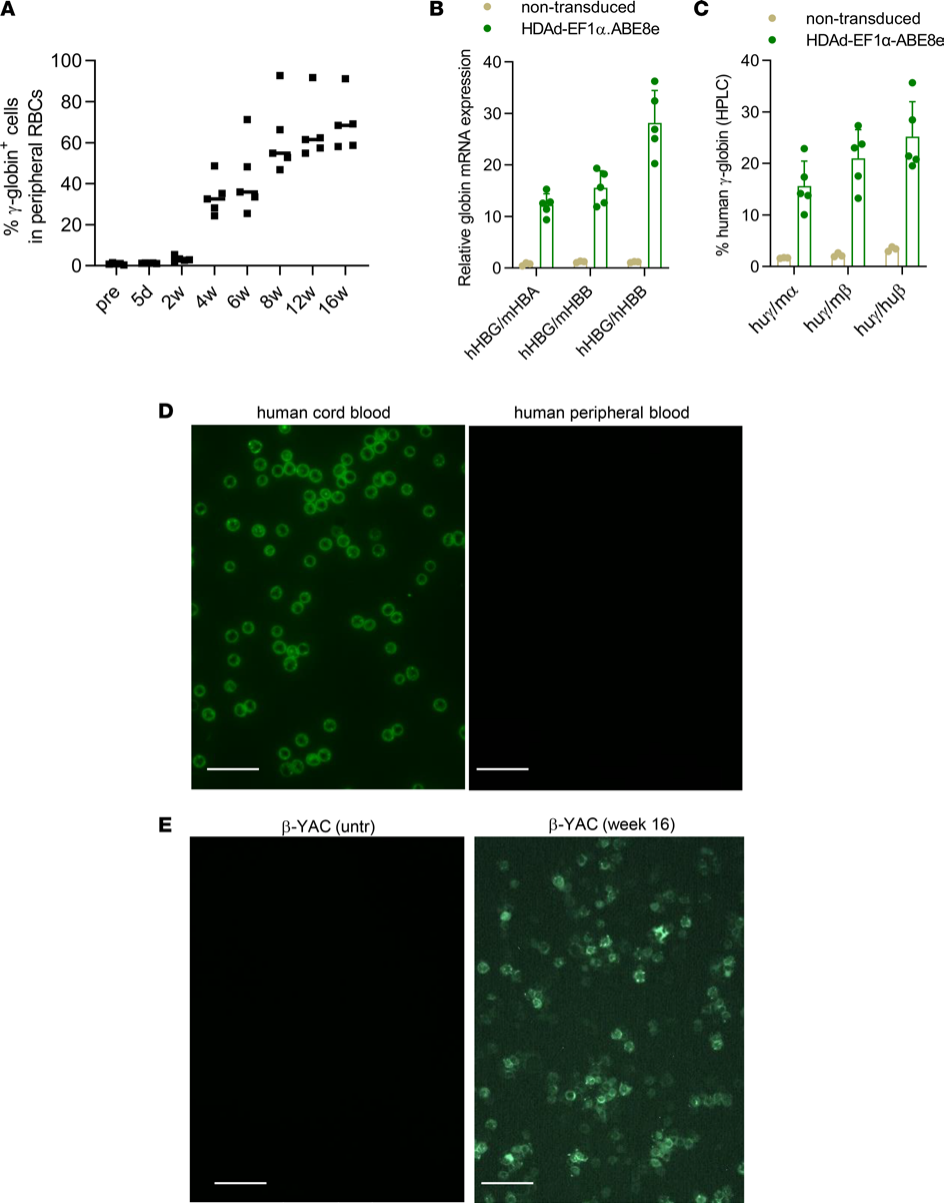
γ-Globin analysis after in vivo base editing with by HDAd-EF1α.ABE8e. (**A**) γ-Globin expression in peripheral RBCs measured by flow cytometry. *n* = 5 animals. (**B**) γ-Globin expression at mRNA level measured by qPCR. Data shown are fold of change over mouse HBA, mHBB, or human HBB mRNA. *n* = 5 animals. (**C**) Human γ-globin chain levels in RBC lysates measured by HPLC. Data shown are percentages over mouse α- or β-globin or human β-globin. *n* = 5 animals. (**D** and **E**) Immunofluorescence analysis of HbF in erythrocytes. (**D**) Human samples. Cytospins of total blood cells from umbilical cord blood (fetus-derived) and from an adult donor. (**E**) Samples from untreated and treaded β-YAC mice (total blood). Cytospins were stained with a GFP-specific antibody. Shown are representative samples out of 5 cytospins per group. Scale bar: 20 μm.

Flow cytometry measuring γ-globin in peripheral RBCs showed a gradual increase from 30% (average at week 4) to 70% γ-globin^+^ RBCs (average at week 16-P) (Figure 8A). A similar kinetic has been observed in our previous studies (12, 38). Levels of human γ-globin (*hHBG*) mRNA relative to human *HBB* mRNA were 30%, on average, at week 16 after in vivo transduction (Figure 8B). Relative to mouse *HBA* and *HBB* mRNA, the level of *hHBG* mRNA was 12% and 15%, respectively. Human γ-globin protein levels, measured by HPLC of erythrocyte lysates, were about 25% of human β-globin chains and approximately 15% or 20% of mouse α- or β-globin chains, respectively (Figure 8C). The presence of HbF in peripheral RBCs of in vivo–treated CD46/β-YAC mice was also shown by staining of cytospins with a human HbF specific antibody (Figure 8, D and E). The signals show a spectrum of intensity, most likely related to how many of the 4 target sites were edited. HbF staining on human peripheral blood and cord blood RBCs were used as negative and positive controls, respectively. Editing rates and percentages of γ-globin^+^ RBCs were maintained in secondary recipients (Supplemental Figure 9).

In summary, in vivo HSC transduction by a single i.v. HDAd injection into mobilized β-YAC/CD46 mice followed by early in vivo selection resulted in > 60% editing at the –113 A site. In total, 25% γ-globin of human β-globin is expressed in > 60% of erythrocytes in healthy mice.

### Off-target editing.

**Figure 9:**
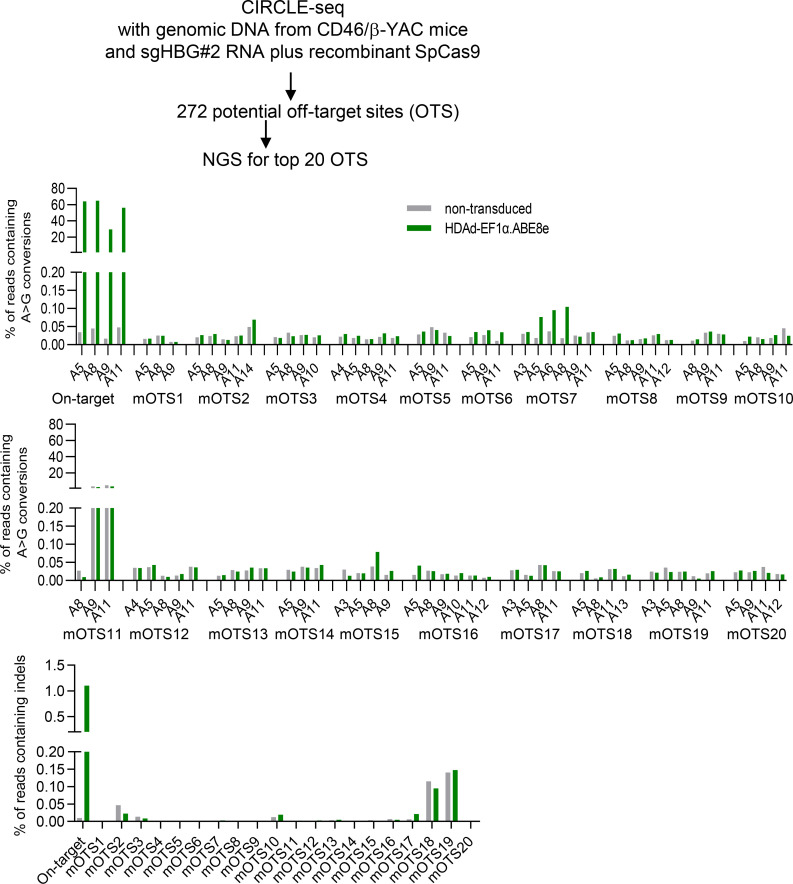
Off-target analysis after in vitro and in vivo base editing with HDAd-EF1α.ABE8e (based on CIRCLE-Seq prediction). Genomic DNA from CD46/β-YAC naive mice was cleaved with recombinant SpCas9 plus sgHBG#2 RNA. CIRCLE-Seq identified 272 OTS, from which the top 20 were subjected to NGS. Amplicons were prepared using a genomic DNA template from a naive mouse and an in vivo transduced mouse with the highest on-targeting editing rate. The middle 2 panels show A>G conversion frequencies in the targetable window (positions 3–14) of the top 20 candidate OTS. Note that, for site mOTS11, the percentage of target-site conversion was similar for untreated and HDAd-EF1α.ABE8e–treated mice, making it unlikely that the conversions were triggered by ABE8e. The bottom panel shows the indel frequency in a quantification window of 140 bp.

**Figure 10:**
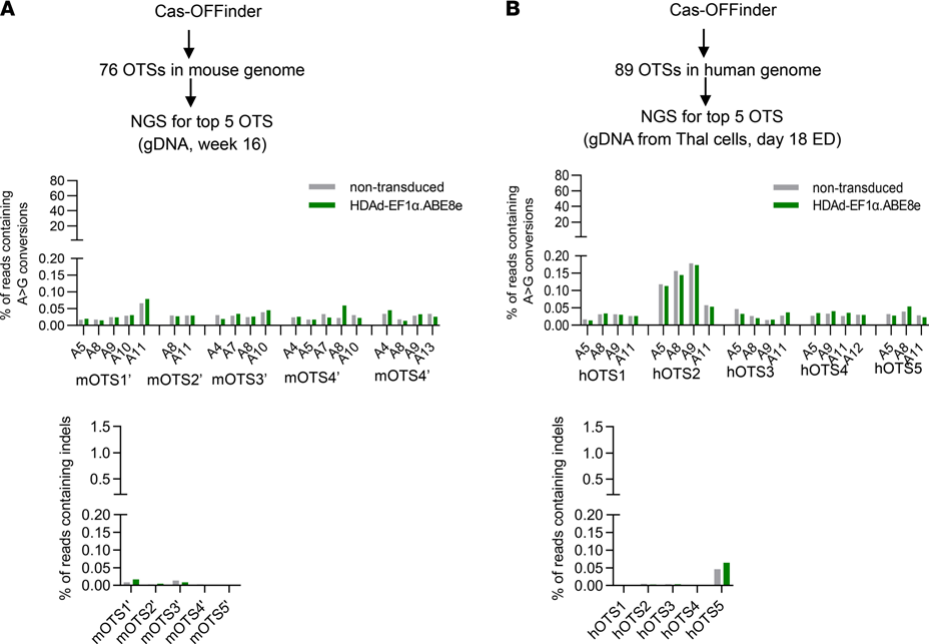
Off-target analysis after in vitro and in vivo base editing with HDAd-EF1α.ABE8e (based on in silico prediction). (**A**) Analysis based on in silico OTS prediction using Cas-OFFinder software for the mouse genome. Seventy-four sites were nominated, from which the top 5 were subjected to NGS. Amplicons were prepared using genomic DNA template from a naive mouse and an in vivo–transduced mouse with the highest on-targeting editing rate. The middle and lower panels show the frequency of A>G conversions and indels, respectively. (**B**) Analysis based on in silico OTS prediction using Cas-OFFinder software for the human genome. Eighty-nine sites were nominated, from which the top 5 were subjected to NGS. Amplicons were prepared using genomic DNA from erythroid cells derived from CD34^+^ cells of a thalassemia patient that were in vitro transduced with HDAd-EF1α.ABE8e.

Using nonintegrating vectors eliminates the risk of insertional mutagenesis. However, the application of genome editing enzymes bears the possibility of genotoxicity due to off-target editing. To assess this, we screened potential off-target editing sites using Circularization for In vitro Reporting of Cleavage Effects by sequencing (CIRCLE-Seq), a highly sensitive in vitro method capable of interrogating genome-wide off-target activity (39) (Figure 9). CD46/β-YAC genomic DNA was cleaved with SpCas9 coupled with sgHBG#2 guide RNA for CIRCLE-Seq. The sgHBG#2 was identical to that used in the HDAd-EF1α.ABE8e vector. CIRCLE-Seq identified a total of 272 candidate off-target sites (OTS) (Supplemental Table 1). To investigate off-target editing in in vivo–transduced mice, we performed targeted deep sequencing for the top 20 of the 272 sites based on the CIRCLE-Seq read count. Genomic DNA from the mouse with the highest on-target editing (65%) (week 16 secondary [week 16-S]) and a naive mouse was used. Results showed that no significant off-target base editing in the editable window (positions 3–14) occurred at these 20 sites (Figure 9, middle panels). Notably, approximately 4% of alleles with single nucleotide variants in OTS #11 were found in both naïve and treated animals. Indel frequencies around the predicted nicking sites (70 bp at each side) of the 20 candidates showed no significant difference between naive and treated animals (Figure 9, bottom panel). We conducted further off-target analyses using Cas-OFFinder, an in silico algorithm to predict potential OTS. A total of 76 and 89 sites with 4 or fewer mismatches to sgHBG#2 guide RNA were nominated in mouse and human genome, respectively (Figure 10, A and B, and Supplemental Tables 2 and 3). Twenty-four of the 76 Cas-OFFinder sites in the mouse genome overlapped with the CIRCLE-Seq OTS list (31.6%). The top 5 ranked sites from each list were amplified from the in vivo–transduced mouse (analyzed above) or in vitro–transduced β-thalassemia CD34^+^ cells for amplicon NGS. Results showed no significant editing at these sites.

### Effect on transcriptome.

**Figure 11:**
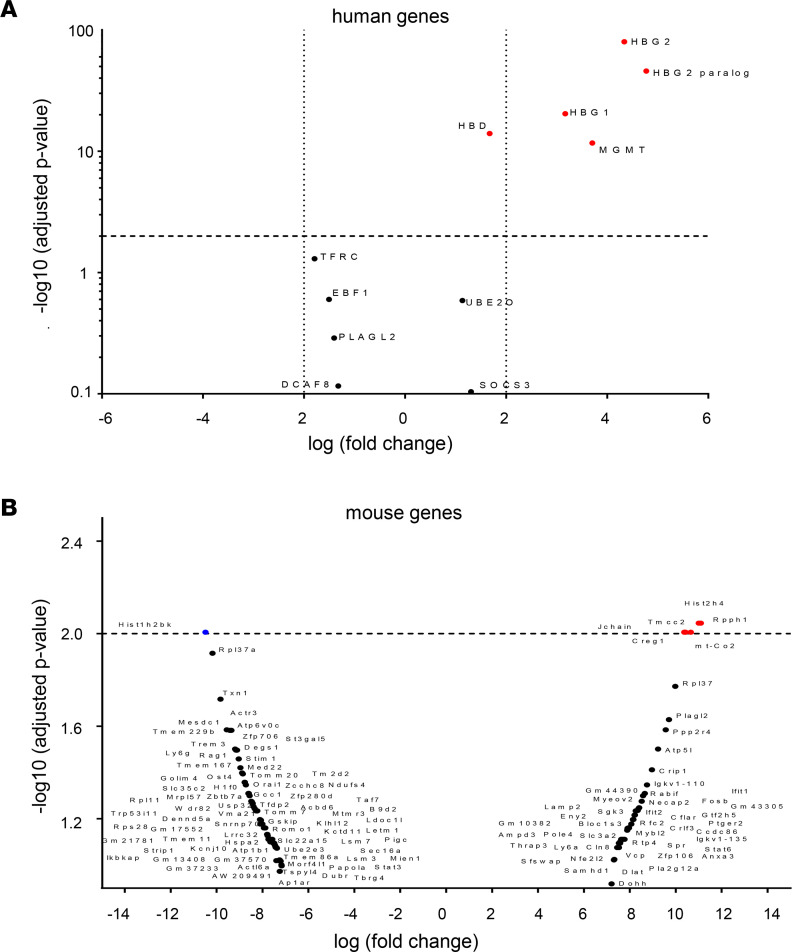
Differentially expressed genes after in vivo base editing HDAd-EF1α.ABE8e. RNA-Seq analysis was performed with BM MNCs collected before treatment and at week 16 after in vivo HSC transduction/selection with HDAd-EF1α.ABE8e (*n* = 3 females, age 8–12 weeks). The genome of CD46/β-YAC mice contains the WT 248 kb β-globin locus (β-YAC) including the LCR, γ*-* and *β-globin* genes. Shown are genes with altered mRNA expression based on their adjusted *P* value after annotation with the human genome. (**A**) Altered mRNA expression after annotation with a reference human genome. Statistically significant changes (*P* < 0.01) are highlighted in red (upregulated). (**B**) Volcano plot of mRNA data after annotation with the mouse reference genome are shown. Statistically significant changes (*P* < 0.01) are highlighted in red (upregulated) or blue (downregulated). Statistical analyses were performed using 2-way ANOVA.

We compared RNA-Seq data of total RNA from BM MNCs of CD46/β-YAC mice before treatment and mice at week 16 after in vivo HSC transduction with HDAd-EF1α.ABE8e (*n* = 3 animals) (Figure 11 and Supplemental Tables 5 and 6) Of the 2,742 genes annotated to the human genome, only 13 were considered differentially expressed (adjusted *P* < 0.01) (Supplemental Table 5). Six of them were upregulated and were clustered based on their biological pathways, into 3 groups: (a) hemoglobin genes, specifically *HBG2, HBG1*, and *HBD*, whose upregulation is most likely the result of in vivo editing of the β-globin locus present in CD46/β-YAC mice, and (b) *mgmt,* as a result of residual expression of human mgmt^P140K^ mRNA from the HDAd vector. Based on the mouse annotation process, we mapped 9,037 genes in total, from which 7 were considered differentially expressed (adjusted *P* < 0.01) (Supplemental Table 6). However, the level of upregulation was barely above the threshold set at 2.0-fold log. Of the 7 genes, 1 gene is listed in the Catalogue of Somatic Mutations in Cancer (COSMIC). This gene, *Creg1*, is upregulated in treated animals. Creg is considered to be a tumor suppressor, and its upregulation should not be associated with cancer (40). Additional studies are required to discover whether the modest upregulation of these genes is due to O^6^-BG/BCNU treatment.

### Hematological analyses.

There were no clinical side effects of the in vivo HSC transduction and selection approaches. Histological analyses of major organs did not show abnormalities. To further assess the safety of in vivo base editing with HDAd-EF1α.ABE8e, we performed hematological analyses of blood and BM samples of week 16 in vivo–transduced CD46/β-YAC mice. No significant differences between treated and untreated mice in blood cell counts (Supplemental Figure 10A), erythroid parameters (Supplemental Figure 10B), platelet counts (Supplemental Figure 10C), and reticulocyte counts (Supplemental Figure 10D) were found.

Furthermore, there were no significant differences in the lineage composition of BM cells (Supplemental Figure 10E). Moreover, in agreement with the disappearance of vector DNA over time, ABE8e was expressed only transiently, which triggered IgM but not a stable IgG anti-Cas antibody response (Supplemental Figure 10F).

In summary, in vivo HSC editing with HDAd-EF1α.ABE8e was found to be safe and yielded γ-globin reactivation at levels that are therapeutically relevant.

## Discussion

The approach described here is aimed toward the reactivation of γ-globin/HbF for the treatment of β-thalassemia and SCD. HbF has powerful antipolymerization properties because its γ-globin subunits form mixed hybrid tetramers of 2 α-globin chains with 1 γ-globin and 1 mutated β-globin chain that are largely excluded from the polymer (41). We used a nonintegrating HDAd5/35++ vector that would trigger permanent genetic edits in HSCs through the transient expression of ABE8e and a sgRNA that targets the distal BCL11A binding motif in the HBG1/2 promoters. Studies were performed in ex vivo and in vivo settings in CD46/β-YAC transgenic mice. The ex vivo approach yielded > 80% HbF^+^ RBCs with γ-globin levels that were 30% of human β-globin levels. Notably, this study was done without any in vivo O^6^-BG/BCNU selection. More importantly, we demonstrated that in vivo HSC transduction of CD46/β-YAC transgenic mice with a single HDAd-EF1α.ABE8e vector and early treatment with O^6^-BG/BCNU resulted in > 60% HbF^+^ RBCs with human γ-globin levels that were 30% of those of human β-globin, in the absence of a competitive background (healthy mice). This outcome is potentially curative in patients with β-thalassemia and SCD. In individuals with HPFH, HbF makes up approximately 30% of total hemoglobin and is homogeneously distributed in the RBC population (42, 43). Furthermore, murine ex vivo gene therapy studies with γ-globin lentivirus vectors demonstrated that long-term amelioration of disease of murine β-thalassemia and SCD occurred when approximately 20% gene-modified HSCs repopulated the marrow, when HbF levels were approximately 20%, and when HbF^+^ cells constituted two-thirds of the circulating RBCs (44–47).

In the absence of β-YAC mice with a thalassemia or SCD genotype or phenotype, we tested disease correction using CD34^+^ cells from patients with β-thalassemia and SCD. We have demonstrated that HDAd-EF1α.ABE8e transduction mediated efficient target site editing and reactivation of γ-globin after ED. This resulted in the reduction of ROS to background level and improved ED/expansion of thalassemia and SCD CD34^+^ cells; it also suggests that the in vivo base editing could be curative in patients with β-thalassemia and SCD.

### Several factors could have contributed to the high efficacy of in vivo editing.

The greatly enhanced catalytic activity of ABE8e (31) and the high level of ABE8e expression in HSC mediated by the EF1α promoter could have contributed. This is supported by comparative studies with HDAd-PGK.ABE8e, where ABE8e expression was driven by the relatively weak PGK promoter and where rates of editing (both ex vivo and in vivo) and γ-globin reactivation were significantly lower (Supplemental Figure 11). The prolonged expression of the base editing machinery from episomal HDAd vector in some transduced HSCs may also have contributed to the high efficacy of in vivo editing. The VCN declined only by 1 order of magnitude within 6 weeks, which implies that the editing machinery is active during that time. This is, in part, supported by the ex vivo study (performed without O^6^-BG/BCNU selection), where an increase of editing rates from the transplant (~38%) to > 95% in week 16 mice was observed. We speculate that the extended presence of the HBG1/2 sgRNA and ABE8e in HSCs could increase the probability for editing of poorly accessible target sites (i.e., sites blocked by heterochromatin at a certain stage of HSC differentiation). In this context, stimulation of cell division by O^6^-BG/BCNU selection could have further increased target site accessibility. As the data in secondary recipients indicate, editing must have occurred in primitive long-term repopulating HSCs (before cell division and differentiation).

Bystander editing of adenines near the –113 target site — specifically –116 A>G, which would destroy the BCL11A binding motif and, most likely, further inhibit the binding of the repressor — may also have contributed to the high efficacy of in vivo editing. The –116 A>G bystander conversion was found in > 99% of –113 A>G edited alleles (Supplemental Figure 12). Also, in the absence of the –110 A>G substitution, a GATA binding motif would be created similar to the –113A>G HPFH (32). This would enable binding of the transcriptional activator GATA1 to the destroyed BCL11A motif and could further increase γ-globin reactivation. Expansion of edited HSPCs with episomal HDAd-EF1α.ABE8e genomes by early treatment with O^6^-BG/BCNU, capitalizing on the prolonged presence of episomal vector genomes, may also have contributed to the high efficacy of in vivo editing. While we did not perform in vivo studies without O^6^-BG/BCNU treatment, the in vitro data with CD34^+^ cells from healthy donors (Figure 1F) and patients with β-thalassemia (Figure 2) illustrate the effect of selection.

Notably, in patients with β-thalassemia and SCD, γ-globin–expressing erythroid cells would have a survival advantage created by the disease background. In this context, we expect that rates of γ-globin reactivation achieved with our approach could be even higher in patients with hemoglobinopathies and the in vivo selection with O^6^-BG/BCNU on demand.

Safety concerns with our approach are related to the i.v. injection of HDAd5/35++ vectors and genotoxicity due to off-target base editing. HDAd vectors are sensed by the RES and can trigger the release/production of proinflammatory cytokines. We have developed pharmacological approaches to avoid these responses in mice (2) and nonhuman primates (NHPs) (8). Furthermore, i.v.-injected HDAd5/35++ vectors preferentially transduce mobilized HSCs (8, 48), minimizing base editing in nonhematopoietic tissues.

Recently, virus-like particles (VLPs) have been used for in vivo editing of murine liver or retina (49). Furthermore, nonviral vehicles based on lipid nanoparticles (LNPs) for mRNA delivery (50, 51) hold great promise for in vivo HSC genome editing. However, these new approaches also face the hurdles accounted with intravascular administration of viral vectors — namely, the unproductive sequestration, which requires high vector doses, and the associated acute toxicity. Furthermore, while viruses have evolved highly efficient mechanisms for cellular uptake and intracellular trafficking, it is not straightforward to incorporate these functions into nonviral delivery vehicles.

Clearly, prolonged expression of the editing machinery could increase the frequency of off-target editing and the genotoxicity risk. Our data indicate that off-target DNA or RNA editing is minimal, while on-target activity is high. No hematological or histological abnormalities were found in primary and secondary mice over a total period of 32 weeks. More than 98% of edits at the target site were the desired A>G conversions; less than 1.5% of reads had small deletions or insertions in the observation window. Interestingly, the indel frequency in secondary mice (at week 16) was significantly lower than in primary mice, indicating a selection against cells with indels over time. NGS of OTS predicted by CIRCLE-Seq or Cas-OFFinder did not show editing in the quantification window. Off-target base editing can manifest as guide-independent, spurious editing of RNA (52, 53) or genomic DNA (31). This, as well as clonal expansion of malignant cells, could result in alterations in the transcriptome. While RNA-Seq shows the expected activation of human γ*-globin* genes (10- to 100-fold log_10_) and residual human mgmt^P140K^ mRNA, changes in the mouse transcriptome were minimal (2.0- to 2.15-fold log_10_), and it is debatable whether these small alterations have biological consequences.

Extensive off-target analyses are crucial for a potential clinical translation of the approach. Therefore, in upcoming NHP studies with an improved HDAd-EF1α.ABE8e vector, great attention will be given to genomic analyses and potential long-term effects of in vivo base editing.

Our current attempts are focused on further improvement of the safety and efficacy of the approach, including new HDAd vector platforms (54), alternative mobilization regimens (55), and in vivo selection strategies. The latter includes an in vivo selection strategy that would not involve low-dose BCNU (56).

We believe that our study is an additional step toward the vision to treat hemoglobinopathies by a simple i.v. injection of a nonintegrating vector.

## Methods

Supplemental Methods are available online with this article.

### CD34+ cell culture

CD34^+^ cells from G-CSF–mobilized healthy adult donors were provided by the Fred Hutch Cell Processing Facility. CD34^+^ cells from patients with β-thalassemia and SCD were previously collected during mobilization clinical trials conducted at George Papanikolaou Hospital, Thessaloniki, Greece (57). CD34^+^ cells were recovered from frozen stocks and incubated overnight in StemSpan H3000 medium (Stemcell Technologies) supplemented with penicillin/streptomycin, Flt3 ligand (Flt3L, 25 ng/mL), IL-3 (10 ng/mL), thrombopoietin (TPO) (2 ng/mL), and stem cell factor (SCF) (25 ng/mL). Cytokines and growth factors were from Peprotech. Cytokines and growth factors were from Peprotech. CD34^+^ cells were transduced with HDAd5/35++ vectors in low-attachment 12-well plates.

### Animal studies

C57BL/6-based transgenic mice that contained the human CD46 genomic locus and provide CD46 expression at a level and in a pattern similar to humans (human CD46tg mice) were described earlier (58). Transgenic mice carrying the WT 248 kb β-YAC were used (23). β-YAC mice were crossed with human CD46tg mice to obtain CD46/β-YAC mice for ex vivo and in vivo studies. The following primers were used for genotyping of mice: *CD46* forward, 5′-GCCAGTTCATCTTTTGACTCTATTAA-3′, and reverse, 5′-AATCACAGCAATGACCCAAA-3′; β-YAC (γ-globin promoter) forward, 5′-AAACGGTCCCTGGCTAAACT-3′, and reverse, 5′-GCTGAAGGGTGCTTCCTTTTT-3′.

#### BM Lin^–^ cell transplantation.

Recipients were 6- to 8-week-old female C57BL/6 mice (The Jackson Laboratory). On the day of transplantation, recipient mice were irradiated (1,000 rad). Four hours after irradiation, 1 × 10^6^ Lin^–^ cells were injected i.v. through the tail vein. This procedure was used for ex vivo HSC transduction studies and for secondary transplantations. The secondary recipients were kept for 16 weeks after transplantation for terminal point analyses.

#### HSC mobilization and in vivo transduction.

HSCs were mobilized in mice by s.c. injection of human recombinant G-CSF (250 μg/kg/mouse/day, 4 days) followed by an s.c. injection of AMD3100 (5 mg/kg) on day 5. In addition, animals received dexamethasone (10 mg/kg, i.p.) 16 hours and 2 hours before virus injection to blunt innate toxicity associated with i.v. HDAd injection. Forty-five minutes after AMD3100, animals were i.v. injected with virus vectors through the retro-orbital plexus (4 × 10^10^ viral particles per mouse).

#### In vivo selection.

Selection was started at day 2 after transduction. Mice were injected with O^6^-BG (15 mg/kg, i.p.) 2 times, 30 minutes apart. One hour after the second injection of O^6^-BG, mice were injected (i.p.) with 5 mg/kg BCNU. At days 12 and 26, 2 more rounds were performed, with BCNU doses at 9 and 10 mg/kg, respectively.

### Data availability

NGS data have been deposited to the NCBI Sequence Read Archive (SRA) with accession no. PRJNA867400.

### Statistics

Data are presented as mean ± SEM. For comparisons of multiple groups, 2-way ANOVA with Bonferroni post hoc testing for multiple comparisons was employed. Statistical analysis was performed using GraphPad Prism version 6.01 (GraphPad Software Inc.). A *P* value less than 0.05 was considered significant.

### Study approval

All experiments involving animals were conducted in accordance with the institutional guidelines set forth by the University of Washington. The University of Washington is an Association for the Assessment and Accreditation of Laboratory Animal Care International–accredited (AALAC-accredited) research institution, and all live animal work conducted at this university is in accordance with the Office of Laboratory Animal Welfare (OLAW) Public Health Assurance (PHS) policy, USDA Animal Welfare Act and Regulations, the *Guide for the Care and Use of Laboratory Animals* (National Academies Press, 2011), and the University of Washington’s IACUC policies. The studies were approved by the IACUC of the University of Washington (protocol no. 3108-01).

## Author contributions

AL and CL provided the conceptual framework for the study. AL, CL, AG, and EN designed the experiments. CL, AG, GAN, KAE, EN, KP, SG, HW, AKA, LH, EV, EY, and TK performed the experiments. DRL and HPK provided critical comments on the manuscript. AL wrote the manuscript.

## Supplementary Material

Supplemental data

Supplemental table 1

Supplemental table 2

Supplemental table 3

Supplemental table 4

Supplemental table 5

Supplemental table 6

## Version 1 08/25/2022

In-Press Preview

## Version 2 10/10/2022

Electronic publication
