# Supplementary material for: In vivo base editing by a single i.v. vector injection for treatment of hemoglobinopathies
Source: JCI Insight. 2022 Oct 10;7(19):e162939. doi: 10.1172/jci.insight.162939 (PMC9675455; doi:10.1172/jci.insight.162939)
Supplement: Supplemental data [file jciinsight-7-162939-s057.pdf]

Ad5/35-Acr-long

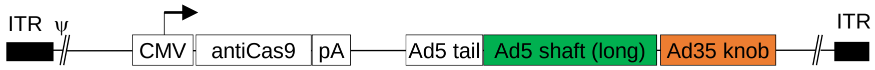

HDAd-ABE8e + Ad5/35-long (no Acr)

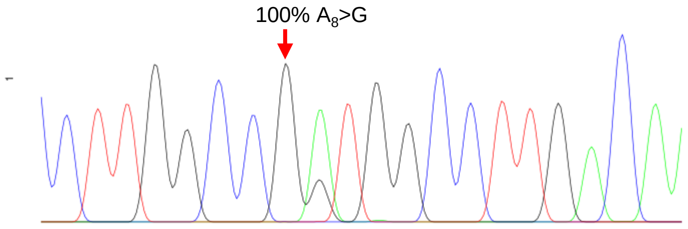

	C	T	T	G	A	C	C	A	A	T	A	G	C	C	T	T	G	A	C	A
T	0	100	100	0	0	0	0	0	0	100	0	0	0	0	100	100	0	0	0	0
G	0	0	0	100	100	0	0	100	27	0	99	100	0	0	0	0	100	0	0	0
C	100	0	0	0	0	100	100	0	0	0	0	0	100	100	0	0	0	0	100	0
A	0	0	0	0	0	0	0	0	73	0	1	0	0	0	0	0	0	100	0	100

HDAd-ABE8e + Ad5/35-Acr-long

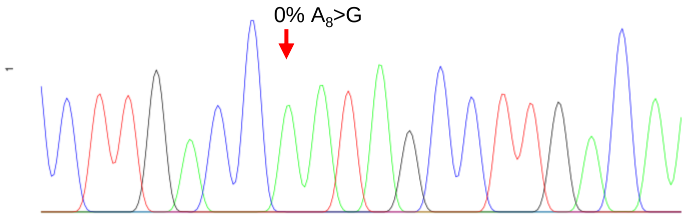

	C	T	T	G	A	C	C	A	A	T	A	G	C	C	T	T	G	A	C	A
T	0	100	100	0	0	0	0	0	0	100	0	0	0	0	100	100	0	0	0	0
G	0	0	0	100	0	0	0	0	0	0	0	100	0	0	0	0	100	0	0	0
C	100	0	0	0	0	100	100	0	0	0	0	0	100	100	0	0	0	0	100	0
A	0	0	0	0	100	0	0	100	100	0	100	0	0	0	0	0	0	100	0	100

Fig.S1. Ad5/35-Acr helper vector to block ABE8e activity in HDAd producer cells (116 cells). Our early attempts to produce the HDAd-EF1α.ABE8e vector failed due to cytotoxicity of high level ABE8e expression in 116 cells. To allow for rescue and high yields of the HDAd-EF1α.ABE8e vector, we produced a new helper virus, Ad5/35-Acr, an Ad5/35++ helper vector containing chimeric fibers composed of the Ad5 fiber tail, the Ad35 fiber shaft, and the affinity-enhanced Ad35++ fiber knob and containing a CMV promoter-AcrI/II expression cassette. We demonstrated that upon co-infection with this helper virus and an HDAd-.ABE8e vector, base editing was completely blocked, most likely by interference of Acr I and II peptides with the interaction between nCas9 and DNA.

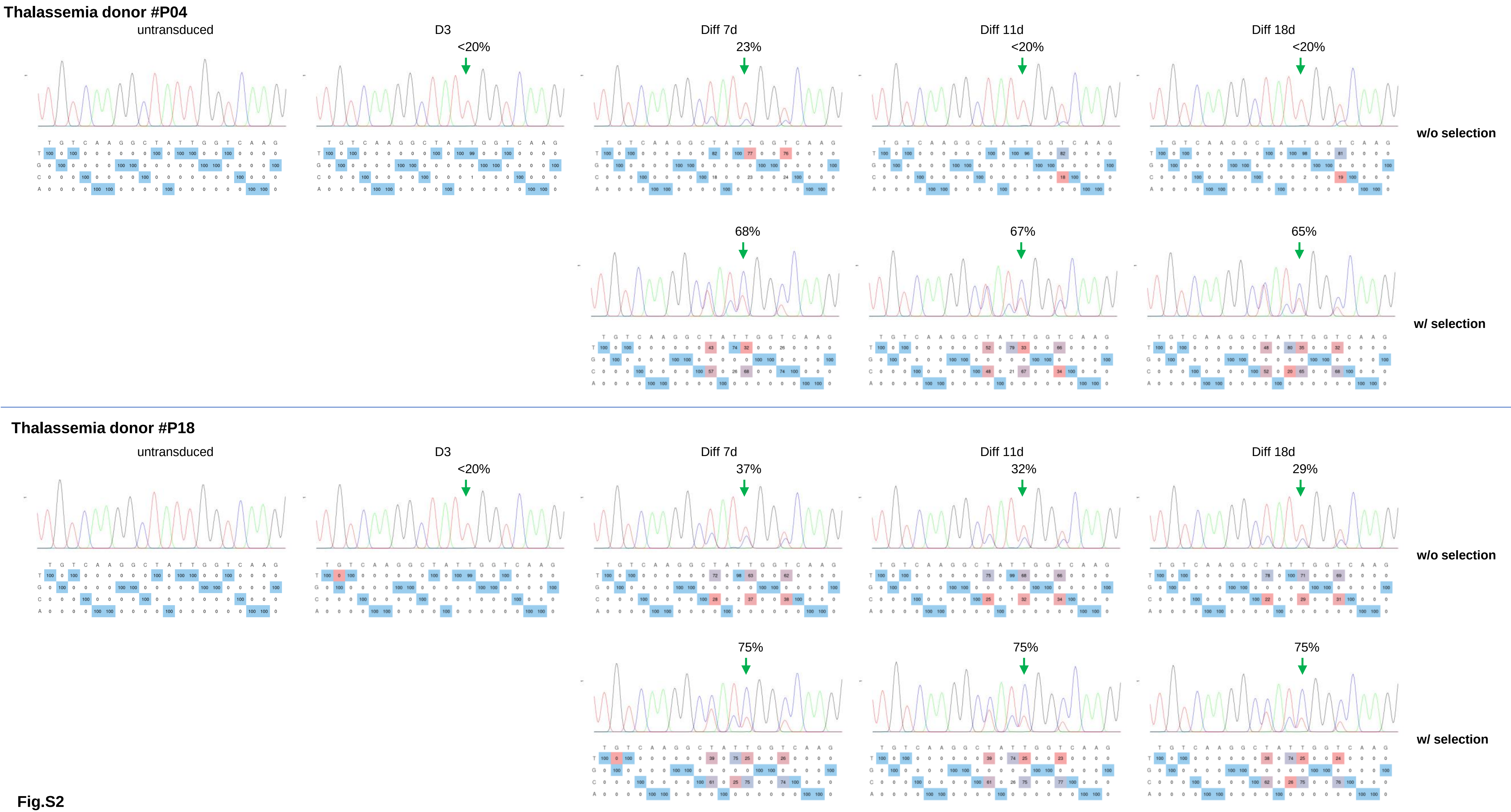

Thalassemia donor #P04
untransduced
D3
<20%
Diff 7d
23%
Diff 11d
<20%
Diff 18d
<20%
w/o selection
68%
67%
65%
w/ selection
Thalassemia donor #P18
untransduced
D3
<20%
Diff 7d
37%
Diff 11d
32%
Diff 18d
29%
w/o selection
75%
75%
75%
w/ selection

Fig.S2

Thalassemia donor #X12

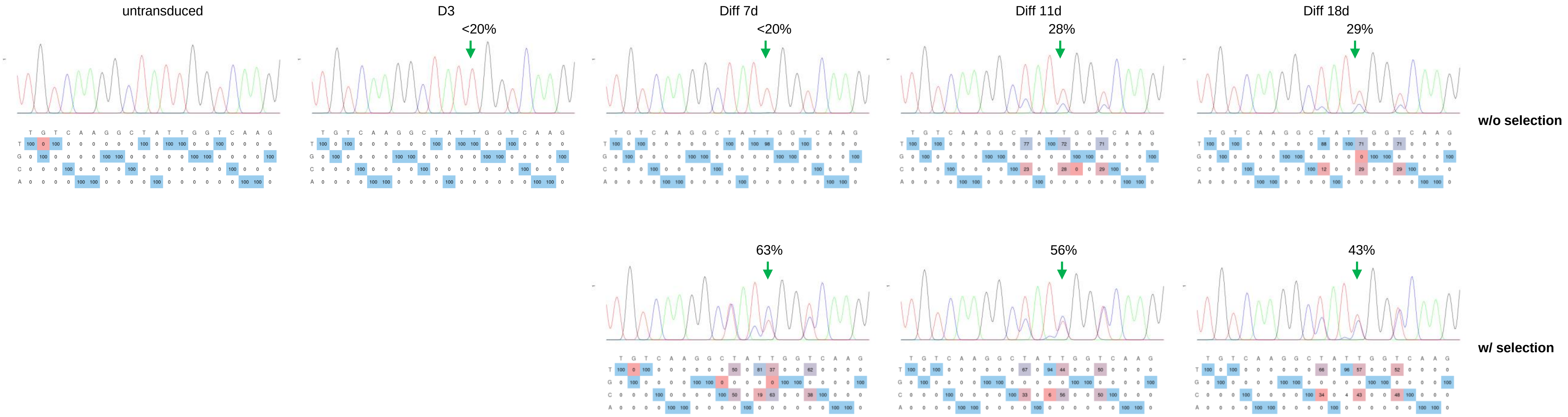

Fig.S2 (continued)

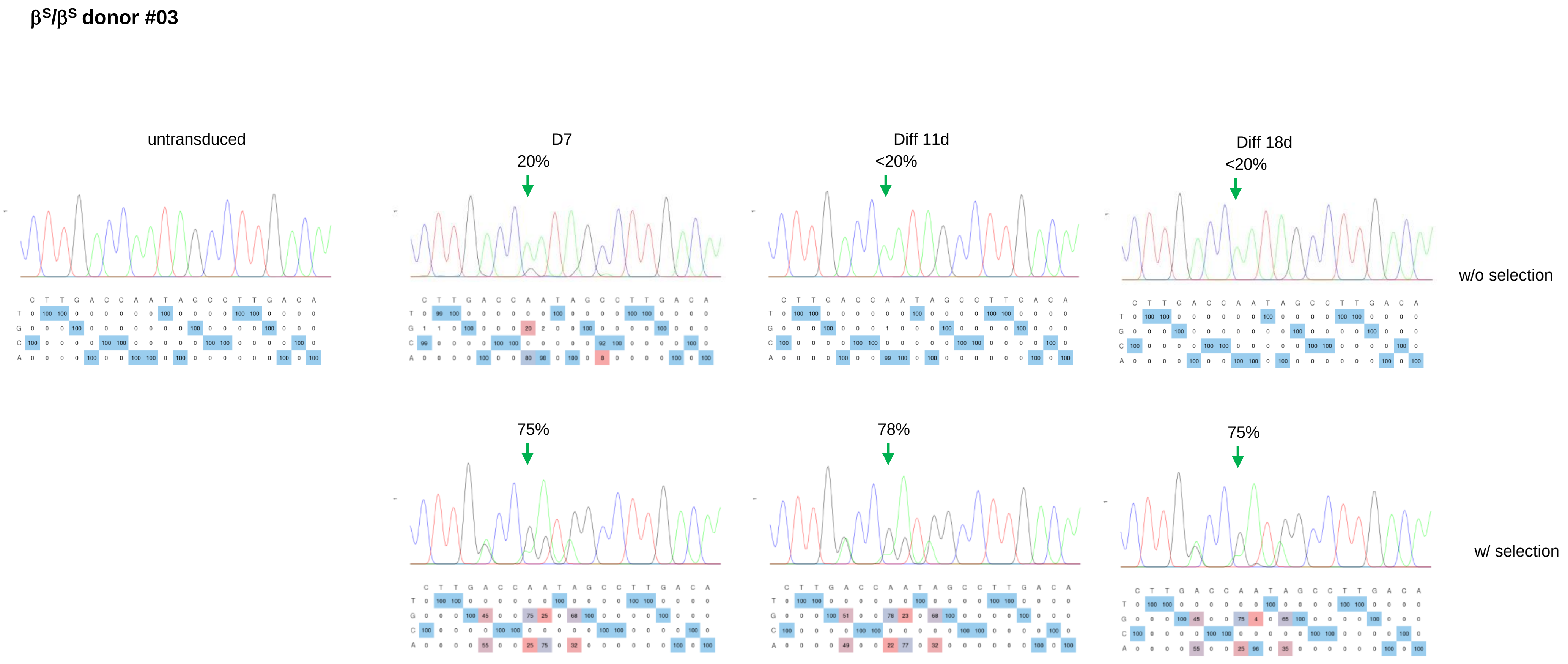

Fig.S2 –continued. *In vitro* base editing of CD34$^+$ cells from three β-thalassemia and one SCD patients measured by Sanger sequencing. Shown are data from untransduced cells and cells that were collected at day 3 after transduction and at days 7, 11, and 18 of ED in settings without and with O^{6}BG/BCNU treatment. Percentages of -113 G>A conversion are indicated. Note, when editing rates are <20%, Sanger sequencing is not accurate.

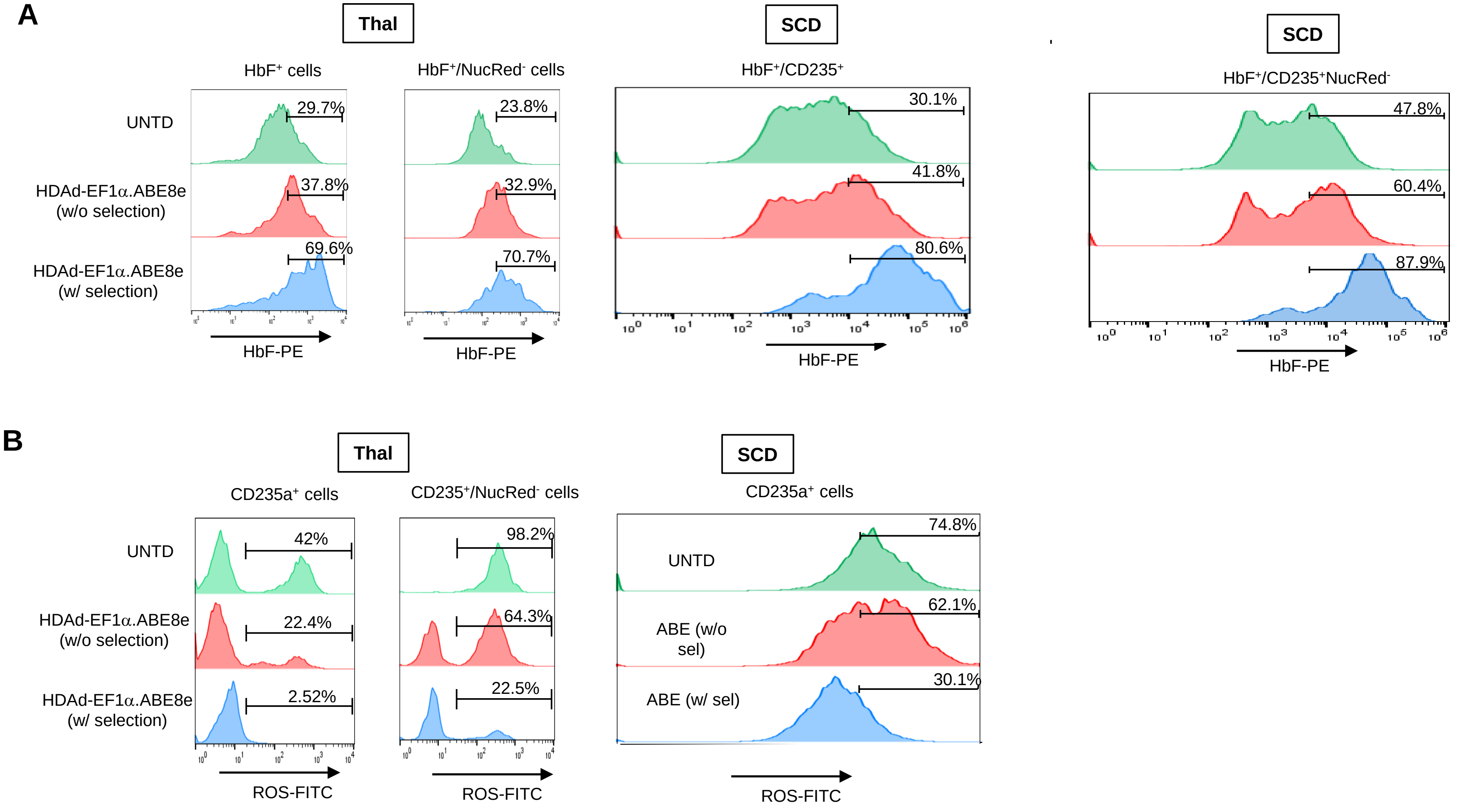

Fig.S3. HbF and ROS flow cytometry analyses. A) Left panels: HbF expression in a representative thalassemia sample at day 18 of ED in total cells and in erythroid (HbF^{+}/NucRed^{-}) cells. Right panels: HbF expression in a representative SCD sample at day 18 of ED in total erythroid (CD235^{+}) cells and in enucleated erythroid (HbF^{+}/CD235a^{+}/NucRed^{-}). **B)** ROS expression in day 18 ED samples. Shown are representative plots corresponding to data from Figs.2C and Fig.2E.

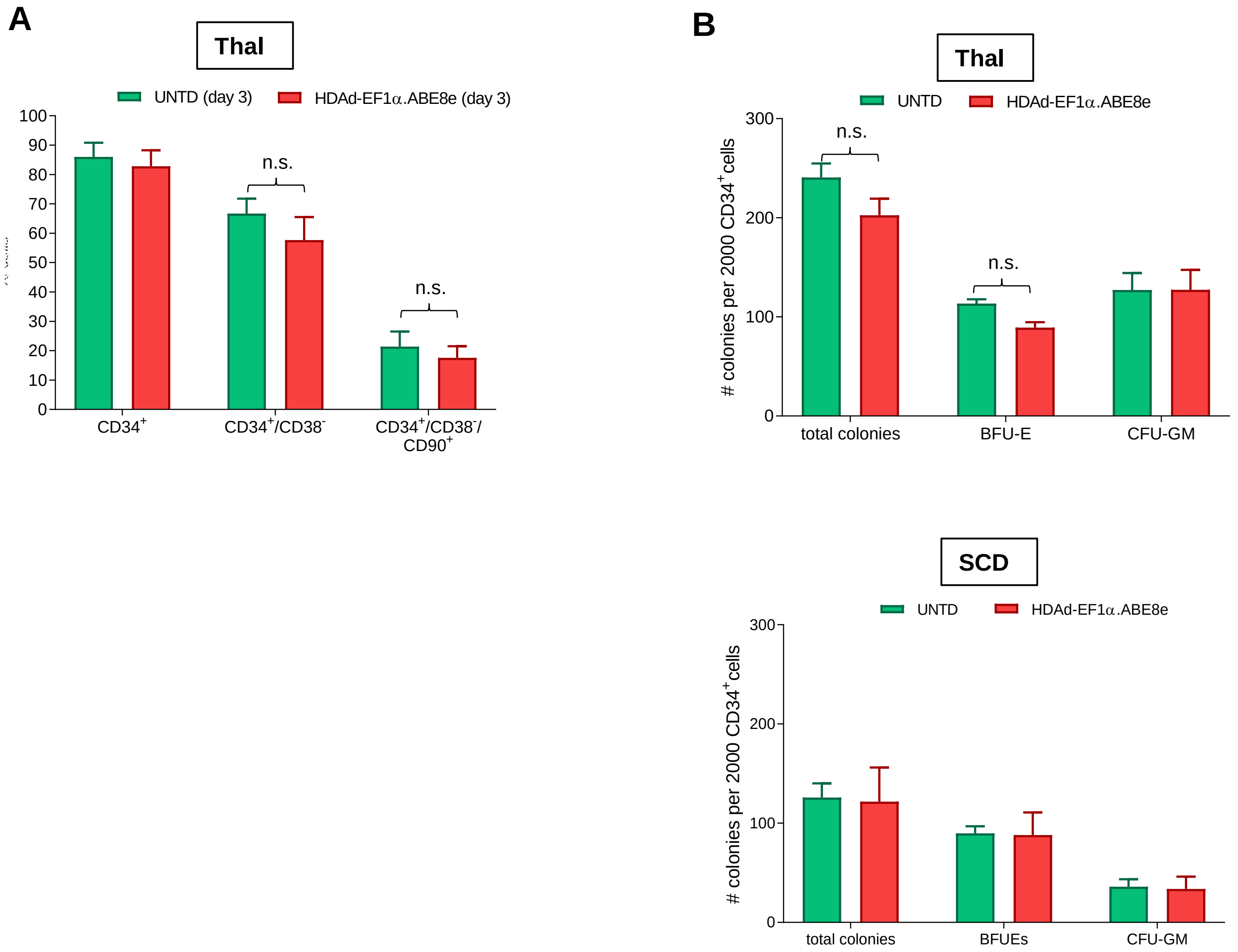

Fig.S4. Absence of cytotoxicity associated with HDAd-EF1α.ABE8e transduction and base editing. A) Percentage of CD34+ cells and more primitive subfractions (CD34+/CD38- and CD34+/CD38-/CD90+) at day 3 of transduction of Thal CD34+ cells. N=3 patients. **B)** Number of colonies counted at day 14 after plating of either untransduced or HDAd-EF1α.ABE8e-transduced CD34+ cells (Upper panel: three thalassemic samples, Lower panel: three SCD patients). Statistical analyses of the data from the Thal samples were performed using two-way ANOVA.

mouse #4207

-113 A>G

Allele sequence	# of reads	% of reads
CCAGCCTTGCCTTGACCAATAGCCTTGACAAGGCAAACTT	12772	7.2
CCAGCCTTGCCTTGGCCGGTGGCCTTGACAAGGCAAACTT	66788	37.9
CCAGCCTTGCCTTGGCCGATGGCCTTGACAAGGCAAACTT	59138	33.53
CCAGCCTTGCCTTGGCCGATAGCCTTGACAAGGCAAACTT	14381	8.15
CCAGCCTTGCCTTGGCCGGTAGCCTTGACAAGGCAAACTT	3467	1.97
CCAGCCTTGCCTTGGCAGATGGCCTTGACAAGGCAAACTT	3229	1.83
CCAGCCTTGCCTTGACCGATGGCCTTGACAAGGCAAACTT	2600	1.47
CCAGCCTTGCCTTGGACGATGGCCTTGACAAGGCAAACTT	1574	0.89
CCAGCCTTGCCTTGACCAATGGCCTTGACAAGGCAAACTT	948	0.54
CCAGCCTTGCCTTGACCA----------CAAGGCAAACTT	671	0.38
CCAGCCTTGCCTTGGCTGGTGGCCTTGACAAGGCAAACTT	650	0.37
CCAGCCTTGCCTTGACCAAG------GACAAGGCAAACTT	575	0.33
CCAGCCTTGCCTTGGCCAATGGCCTTGACAAGGCAAACTT	553	0.31
CCAGCCTTGCCTTGGTCGATGGCCTTGACAAGGCAAACTT	483	0.27
CCAGCCTTGCCTTGACCGATAGCCTTGACAAGGCAAACTT	477	0.27
CCAGCCTTGCCTTGACCGGTGGCCTTGACAAGGCAAACTT	472	0.27
CCAGCCTTGCCTTGGCAGGTGGCCTTGACAAGGCAAACTT	470	0.27
CCAGCCTTGCCTTGGCTGATGGCCTTGACAAGGCAAACTT	409	0.23
CCAGCCTTGCCTTGACCGA----------AAGGCAAACTT	402	0.23
CCAGCCTTGCCTT------TGGCCTTGACAAGGCAAACTT	339	0.19

mouse #4208

Allele sequence	# of reads	% of reads
CCAGCCTTGCCTTGACCAATAGCCTTGACAAGGCAAACTT	13646	7.0
CCAGCCTTGCCTTGGCCGATGGCCTTGACAAGGCAAACTT	73591	37.7
CCAGCCTTGCCTTGGCCGGTGGCCTTGACAAGGCAAACTT	61393	31.45
CCAGCCTTGCCTTGGCCGATAGCCTTGACAAGGCAAACTT	24047	12.32
CCAGCCTTGCCTTGGCCGGTAGCCTTGACAAGGCAAACTT	3013	1.54
CCAGCCTTGCCTTGGCTGATGGCCTTGACAAGGCAAACTT	2720	1.39
CCAGCCTTGCCTTGACCGATGGCCTTGACAAGGCAAACTT	2277	1.17
CCAGCCTTGCCTTGGCAGATGGCCTTGACAAGGCAAACTT	1788	0.92
CCAGCCTTGCCTTGACAGATGGCCTTGACAAGGCAAACTT	867	0.44
CCAGCCTTGCCTTG------------GACAAGGCAAACTT	808	0.41
CCAGCCTTGCCTTGGCGGATGGCCTTGACAAGGCAAACTT	726	0.37
CCAGCCTTGCCTTGGCAGGTGGCCTTGACAAGGCAAACTT	697	0.36
CCAGCCTTGCCTTGGCCCATGGCCTTGACAAGGCAAACTT	606	0.31
CCAGCCTTGCCTTGGCAGATAGCCTTGACAAGGCAAACTT	544	0.28
CCAGCCTTGCCTTGACCGGTAGCCTTGACAAGGCAAACTT	473	0.24
CCAGCCTTGCCTTGGCCAATAGTGGCCTTGACAAGGCAAACTT	409	0.21
CCAGCCTTGCCTTGGCTGGTGGCCTTGACAAGGCAAACTT	407	0.21
CCAGCCTTGCCTTGACCGATAGCCTTGACAAGGCAAACTT	389	0.20
GCAGCCTTGCCTTGGCCGATGGCCTTGACAAGGCAAACTT	327	0.17
CCAGCCTTGCCTTGGCCAATGGCCTTGACAAGGCAAACTT	260	0.13

mouse #4209

Allele sequence	# of reads	% of reads
CCAGCCTTGCCTTGACCAATAGCCTTGACAAGGCAAACTT	18504	8.7
CCAGCCTTGCCTTGGCCGATGGCCTTGACAAGGCAAACTT	77540	36.5
CCAGCCTTGCCTTGGCCGGTGGCCTTGACAAGGCAAACTT	70236	33.10
CCAGCCTTGCCTTGGCCGATAGCCTTGACAAGGCAAACTT	21491	10.13
CCAGCCTTGCCTTGGCCGGTAGCCTTGACAAGGCAAACTT	2355	1.11
CCAGCCTTGCCTTGACCGATGGCCTTGACAAGGCAAACTT	2137	1.01
CCAGCCTTGCCTTGACCAAT------GACAAGGCAAACTT	2074	0.98
CCAGCCTTGCCTTGGCAGATGGCCTTGACAAGGCAAACTT	1772	0.84
CCAGCCTTGCCTTGACCAATGGCCTTGACAAGGCAAACTT	1516	0.71
CCAGCCTTGCCTTGGCC-----------------(34bp)-TT	1481	0.70
CCAGCCTTGCCTTGGCTGATGGCCTTGACAAGGCAAACTT	1409	0.66
CCAGCCTTGCCTTGGCCAATGGCCTTGACAAGGCAAACTT	1020	0.48
CCAGCCTTGCCTTGGCAGGTGGCCTTGACAAGGCAAACTT	870	0.41
CCAGCCTTGCCTTGACCAAGATGGCCTTGACAAGGCAAACTT	642	0.30
CCAGCCTTGCCTTGGCGGGTGGCCTTGACAAGGCAAACTT	569	0.27
CCAGCCTTGCCTTG-------GCCTTGACAAGGCAAACTT	534	0.25
CCAGCCTTGCCTTGGACGGTGGCCTTGACAAGGCAAACTT	520	0.25
CCAGCCTTGCCTTGACCGATAGCCTTGACAAGGCAAACTT	518	0.24
CCAGCCTTGCCTTGGCTGGTGGCCTTGACAAGGCAAACTT	324	0.15
CCAGCCTTGCCTTGGTCGGTGGCCTTGACAAGGCAAACTT	310	0.15

Fig.S5. NGS analysis of bone marrow MNCs collected at week 16-P after transplantation in the *ex vivo* study shown in Fig.5.

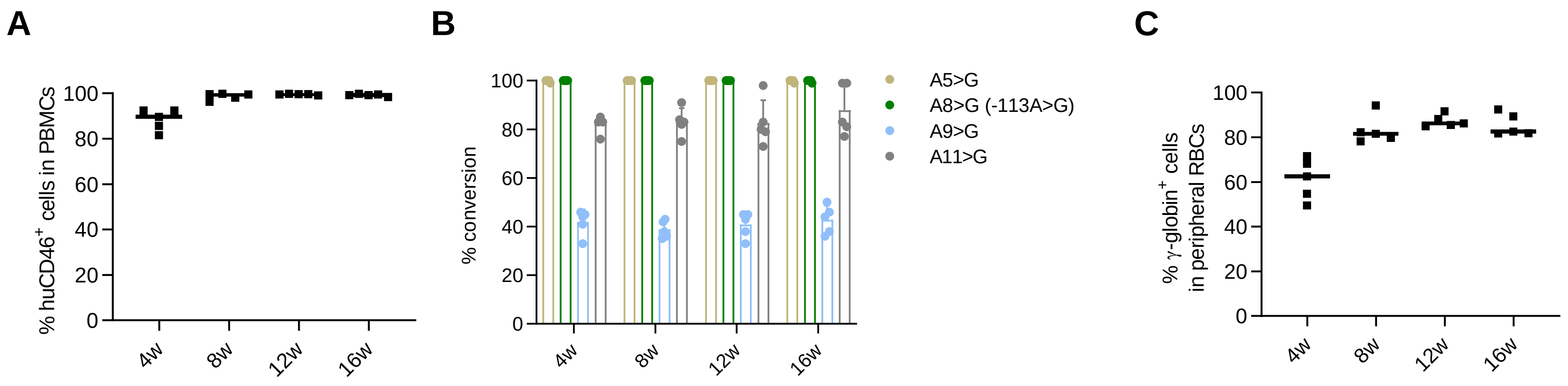

Fig.S6. Analysis of secondary recipients for the *ex vivo* HSC transduction study shown in Figs5 and 6. A) Engraftment of transplanted HDAd-EF1α.ABE8e-transduced HSCs measured by flow cytometry of human CD46 in PBMCs. Each symbol is an individual mouse. N=5 animals. **B)** Analysis of target site editing in PBMCs by Sanger sequencing. Shown are percentages of conversion for the -113A>G site and neighboring adenines. N=5 animals. **C)** Percentage of γ-globin positive peripheral red blood cells (RBCs) measured by flow cytometry. N=5 animals.

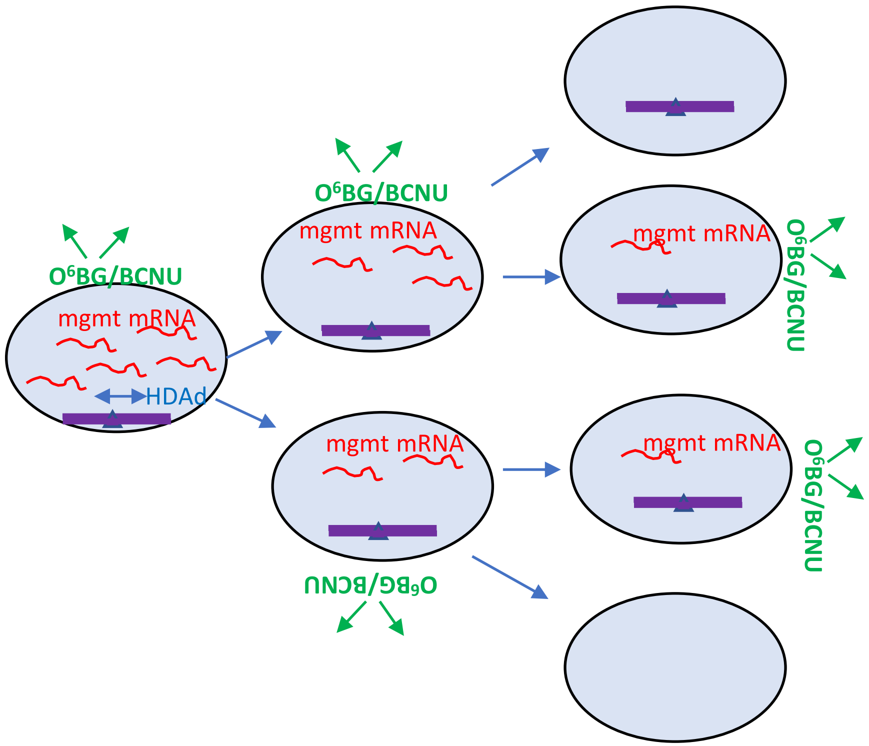

Fig.S7. Expansion of edited cells based on mgmt^{P140K} expression from episomal HDAd-EF1α.ABE8e vector genomes. Concept of O^{6}BG/BCNU selection with non-integrating HDAd vectors. After HDAd-EF1α.ABE8e transduction of HSCs, mgmt^{P140K} mRNA and protein are expressed which confers resistance to O^{6}BG/BCNU and selective expansion of transduced cells. Upon cell division, HDAd vector genomes, mgmt^{P140K} mRNA, and protein a gradually lost.

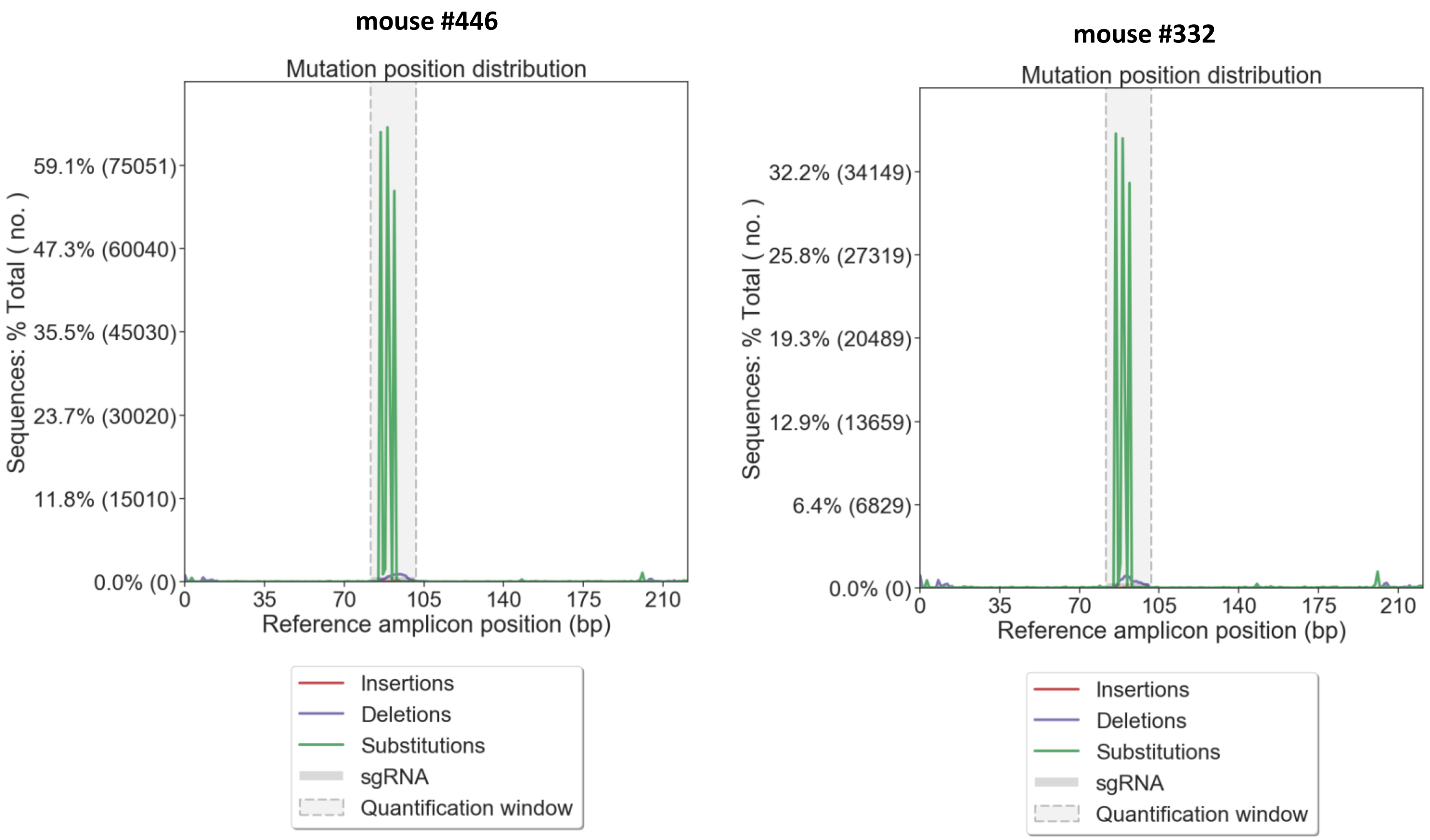

Fig.S8. Characterization of nucleotide modifications at the target sites in two representative mice (Bone marrow MNCs at week 16). See also Fig.7G.

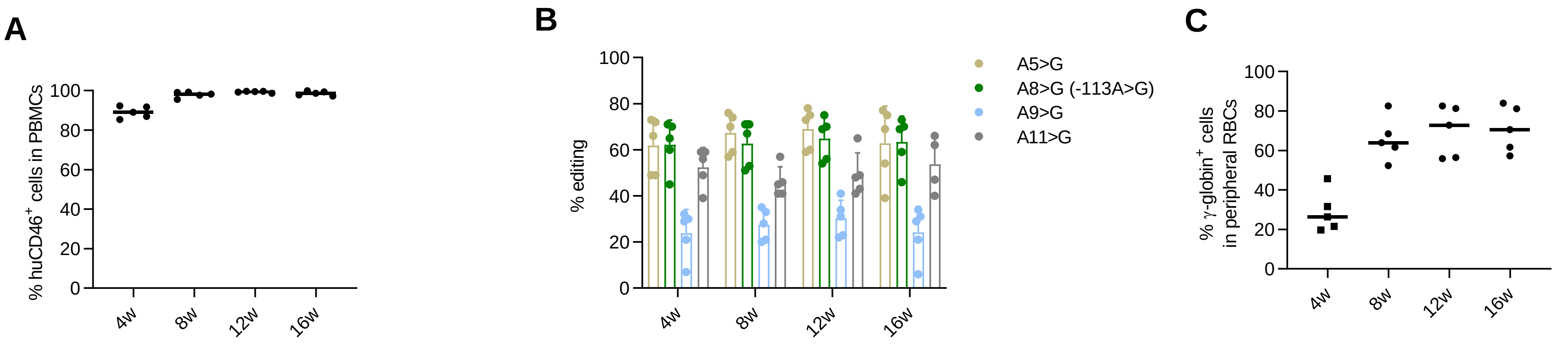

Fig.S9. Secondary recipients of Lin- cells from *in vivo* transduced mice shown in Figs.7 and 8. A) Engraftment based on the percentage of huCD46+ cells in PBMCs. N=5 animals. **B)** Editing rate in PBMCs measured by Sanger sequencing. **C)** Percentage of γ-globin+ RBCs in peripheral blood measured by flow cytometry. N=5 animals.

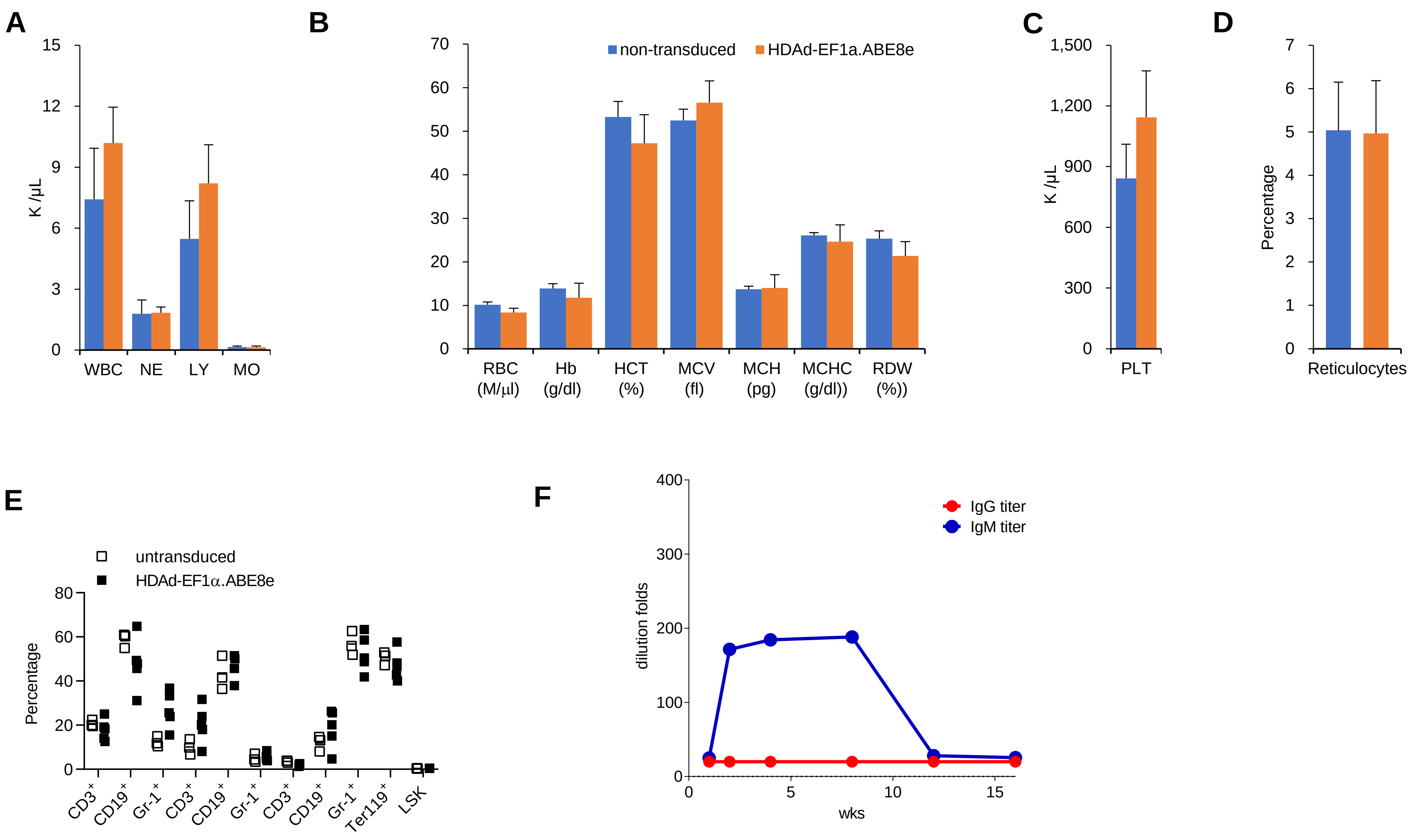

Fig.S10. Safety profile. **A**) Hematology analysis using blood samples collected at week 16 after *in vivo* transduction. Data shown are mean ± SD representing five mice transduced with HDAd-EF1α.ABE8e and three non-transduced naive mice. **B**) Erythroid cell parameters. HCT: hematocrit, MCH: Mean Corpuscular Volume, MCH: Mean Corpuscular Hemoglobin, MCHC: Mean Corpuscular Hemoglobin Concentration, RDW: Red Cell Distribution Width. **C)** Platelet counts. **D)** Percentage of reticulocytes in peripheral blood. **E)** Lineage composition of bone marrow mononuclear cells. N=3 for non-transduced animals. **F)** Serum anti-Cas9 IgM and IgG titers based on IC_{50} (average of two mice).

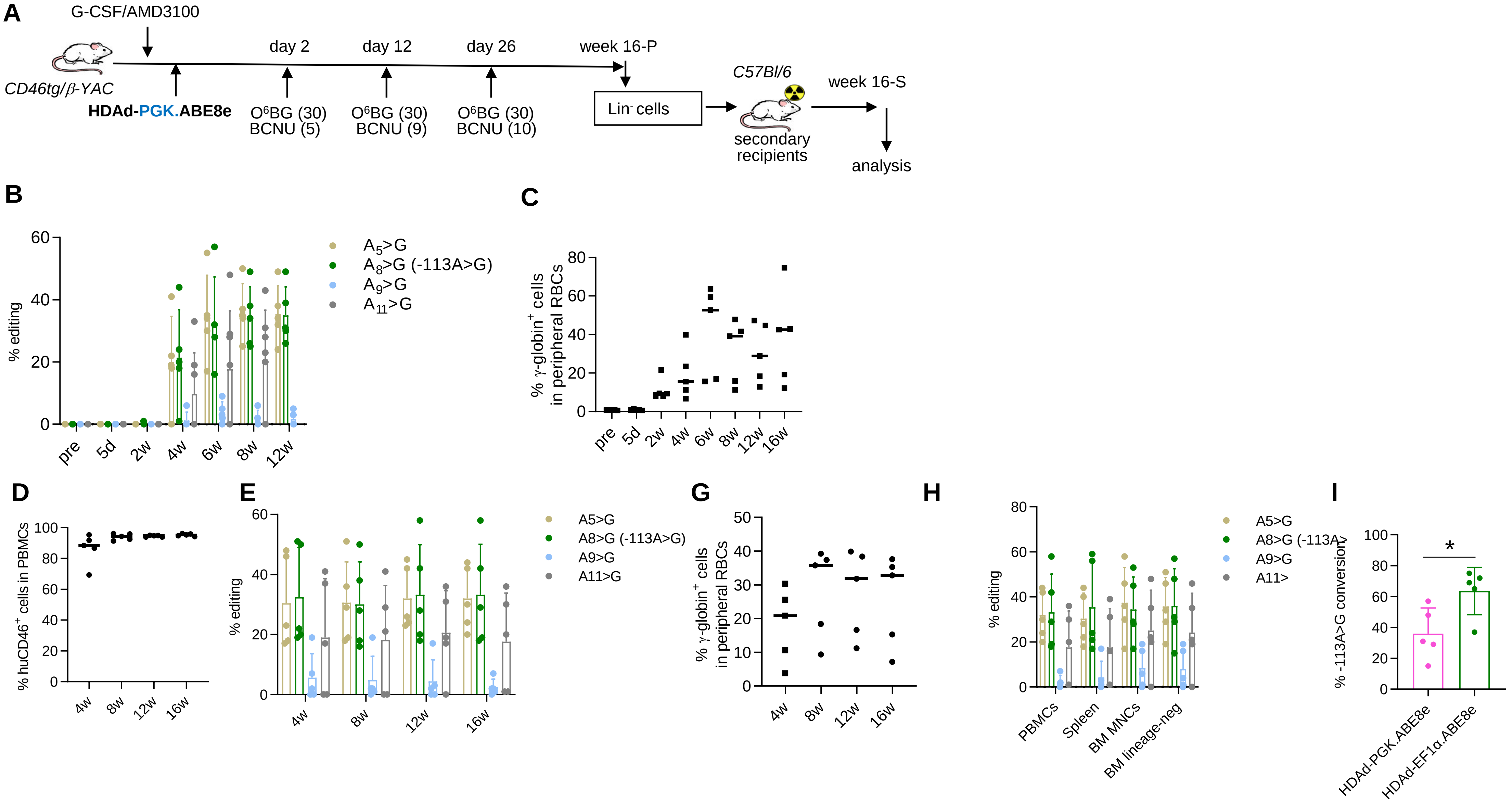

Fig.S11. *In vivo* HSC transduction of β-YAC/CD46 mice to achieve γ-globin reactivation by HDAd-PGK.ABE8e. A) The experimental procedure is the same as described in Fig.7A, except that the HDAd-ABE8e vector contained the base editor under the control of the PGK promoter. **B and C)** Analyses of *in vivo* transduced mice. **B)** Target base conversion measured by Sanger sequencing. Each dot represents one animal. N=5 animals. **C**) γ-globin expression in peripheral red blood cells (RBCs) measured by flow cytometry N=5. **D-H)** Analysis of secondary recipients. N=5 animals. **D)** Engraftment based the percentage of huCD46+ cells in PBMCs. **E)** Editing rate in PBMCs measured by Sanger sequencing. **G)** Percentage of γ-globin+ RBCs in peripheral blood measured by flow cytometry. **H)** Percent conversion at week 16 in PBMCs and mononuclear cells in the spleen and bone marrow as well as in bone marrow Lin- cells. **I)** Comparison of editing rates in mice *in vivo* transduced with HDAd-PGK.ABE8e versus HDAd-EF1α.ABE8e. Week 16 bone marrow MNC samples from secondary recipients were used. N=5 animals. *, $p<0.05$. Statistical analyses in panel I) were performed using two-way ANOVA.

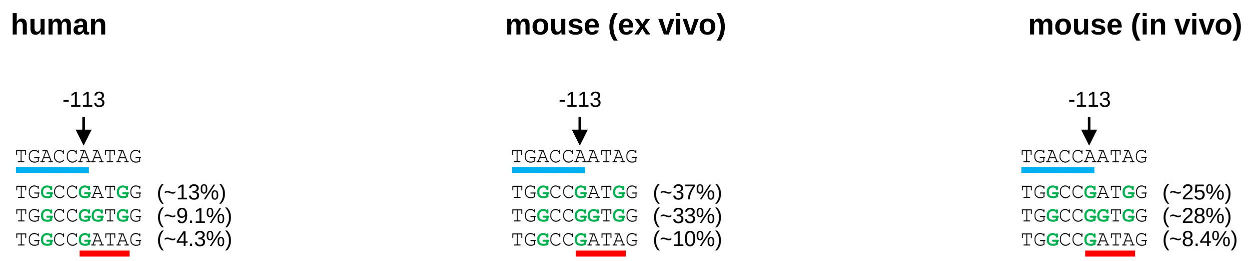

Fig.S12. Bystander adenine editing. Green letters indicate edited nucleotides. The BCL11A binding motif is underline in blue. The newly generated GATA binding site is underlined red. In parentheses are percentage of reads.

Supplementary Methods

Reagents for *in vivo* transduction and selection: G-CSF (Neupogen™) (Amgen, Thousand Oaks, CA), AMD3100 (MilliporeSigma, Burlington, MA), and Dexamethasone Sodium Phosphate (Fresenius Kabi USA, Lake Zurich, IL) were used. O^6-Benzylguanine (O^6BG) and Carmustine (BCNU) were from Millipore/Sigma.

Generation of HDAd vectors: HDAd5/35++ mgmt/GFP has been described earlier (1). We used the ABEmax and ABE8e base editing systems developed by David R. Liu's lab at Harvard University. The oligonucleotides and gBlocks described below were synthesized by Integrated DNA Technologies (IDT) (Coralville, IA) and listed in supplementary Table S4.
First version of ABE8e construct (ABE8e.v1 with a PGK promoter): The cloning involved three steps. Step 1) A 391 bp fragment was PCR amplified from pBS-ABEopti-sgHBG#2-miR with primers #1FR; A 498 bp gBlock (#2) containing the Tad8e of ABE8e sequence was synthesized; for primers, see Supplemental Table 4. A 2.0 kb fragment was PCR amplified from pBS-ABEmax-sgHBG#2-miR with primers #3FR. The three fragments were combined with *SpeI/EcoRV*-digested pBS-ABEopti-sgHBG#2-miR, resulting in pBS-ABE8e-sgHBG#2-miR. Step 2) A 0.6 kb 1gmt. fragment (from *NotI* to *AatII*) was amplified from pHCAS2-MCS-PGK-mgmt (described below) using primers #4FR, followed by infusion with *NotI/AatII*-digested pHCAS3-EF1α-mgmtGFP-, generating pHCAS3-EF1α-mgmt-MCS. Step 3) The two products from step 1 and 2 were digested with *PacI* and recombined with infusion cloning, generating pHCA-PGK-ABE8e-sgHBG#2-v1.
Second version of ABE8e construct (ABE8e.v3 with an EF1α promoter): The cloning involved three steps. Step 1) A 1.5kb fragment was PCR amplified from pBS-ABEmax-sgHBG#2-miR with primers #5FR; The #2 gBlock and 2.0 kb fragment were described above. These three fragments were joined together with *AscI/EcoRV*-digested pBS-ABEmax-sgHBG#2-miR using infusion cloning, forming pBS-ABE8e-sgHBG#2-miR-v2. An 0.3 kb fragment with the vaRNA_miR183/218_vaRNA cassette was PCR amplified from pBS-CMVGFP-miRNA(2) using #6FR and then inserted at the *NotI* site of pBS-ABE8e-sgHBG#2-miR-v2, generating to pBS-ABE8e-sgHBG#2-miR-v3. Step 2) A 6.1 kb fragment containing the pBS vector backbone and the PGK-mgmt cassette was amplified from pBS-Frt-IR-LCR-globin-PGK-mgmt using #7FR, followed by NdeI digestion of the amplicon and then self-circularization by T4 ligase, obtaining pBS-PGK-mgmt. A 3.2 kb PGK-mgmt fragment amplified from pBS-PGK-mgmt using primers #8FR was then ligated with *SwaI/PshAI*-digested pHCAS2-MCS, generating pHCAS2-MCS-PGK-mgmt. Next, a 1.6 kb stuffer DNA amplified from pHCA using #9FR was inserted at the *SwaI* site of pHCAS2-MCS-PGK-mgmt, getting pHCAS3-MCS-PGK-mgmt. Step 3) The resultant constructs from step 1 and 2 were digested with *PacI* and recombined with infusion cloning, generating pHCA-EF1α-ABE8e-sgHBG#2-v3.
The Phusion Hot Start II High-Fidelity DNA Polymerase (New England Biolabs) was used in all PCR amplifications involved in cloning. Final constructs were screened by several restriction enzymes (*HindIII*, *EcoRI* and *PmeI*) and confirmed by sequencing the whole region containing transgenes.
pAd5/35L-Acr helper construct: The pAd5/35 long-shafted helper plasmid was linearized with SrfI and then SpCas9 (New England Biolabs, Cat# M0386S) coupled with guide sequence #10 by following instructions of the EnGen® sgRNA Synthesis Kit, S. pyogenes (New England Biolabs, Cat# E3322V). The >35 kb band was purified and recombined with a 2.2 kb fragment amplified from pAd5/35 helper using #11FR and a 1.8 kb fragment amplified from pNG163-CMV-antiCas9-A using #12FR, generating pAd5/35L-Acr containing a long-shafted 5/35 fiber and the anti-CRISPR sequence.
Helper virus was produced by transfecting low-passage HEK293 cells. For the production of HDAd5/35++ vectors, corresponding plasmids were linearized with *PmeI* and rescued in 116 cells with HD-Ad5/35++ vectors were amplified in 116 cells as described in detail elsewhere Helper virus contamination levels were found to be <0.05%. Titers were 1-5x10^{12} viral particles (vp)/mL.

***In vitro* erythroid differentiation of CD34$^+$ cells with O^{6}BG/BCNU selection.** Differentiation of human CD34$^+$ cells into erythroid cells was done based on the protocol developed by Douay *et al*. In brief, in step 1, cells at a density of 10^4 cells/ml were incubated for 7 days in IMDM supplemented with 5% human plasma, 2 IU/ml heparin, 10μg/ml insulin, 330 μg/ml transferrin, 1 μM hydrocortisone, 100 ng/ml SCF, 5 ng/ml IL-3, 3 U/ml erythropoietin (Epo), glutamine, and penicillin/streptomycin. In step 2, cells at a density of $1x10^5$ cells/ml were incubated for 3 days in IMDM supplemented with 5% human plasma, 2 IU/ml heparin, 10 μg/ml insulin, 330 μg/ml transferrin, 100 ng/ml SCF, 3 U/ml Epo, glutamine, and Pen/Strep. In step 3, cells at a density of $1x10^6$ cells/ml cells were incubated for 12 days in IMDM supplemented with 5% human plasma, 2 IU/ml heparin, 10 μg/ml insulin, 330 μg/ml transferrin, 3 U/ml Epo, glutamine, and Pen/Strep. For the enrichment of transduced cells, 48 hours post-transduction, CD34$^+$ cells were treated with 50μM O^{6}BG and 25-35 μM BCNU. Specifically, cells were incubated with 50μM fresh O^{6}BG for one hour. Without washing fresh 35μM BCNU were added for 2 hours, after which, cells were washed and resuspended in fresh medium.

Blood analyses: Blood samples were collected into EDTA-coated tubes and analysis was performed on a HemaVet 950FS (Drew Scientific, Waterbury, CT). Peripheral blood smears were stained with Giemsa/May-Grünwald (Merck, Darmstadt, Germany) for 5 and 15 minutes, respectively.

Colony-forming unit (CFU) assay: Lineage minus (Lin$^-$) cells were isolated by depletion of lineage-committed cells in bone marrow MNCs using the mouse lineage cell depletion kit (Miltenyi Biotec, San Diego, CA) according to the manufacturer's instructions. CFU assays were performed using ColonyGEL 1202 (Reachbio, Seattle, WA) with mouse complete medium according to the manufacturer's protocol. Colonies were scored 10 days after plating. For human CD34$^+$ cells, ColonyGEL 1102 (Reachbio, Seattle, WA) with human complete medium was used. Colonies derived from human HSPCs were counted at day 14.

Measurement of base conversion by Sanger sequencing and Next-Generation Sequencing (NGS): For Sanger sequencing, genomic segments encompassing the target sites were amplified using primers: *HBG1* forward, 5'-CACACTCCACACTTTTTTGTTTAC-3', reverse, 5'-AAGTGTCTTTACTGCTTTTATTTGCT-3'; *HBG2* forward, 5'-TCCTTCTGTCATTTTGCCTCTGTT-3', reverse, 5'-CACTTCATTGTAGTTACCGTGGAAAGA-3'; *HBG1/2* forward, 5'-GACGTTCCAGAAGCGAGTGT-3', reverse, 5'-CCCTTCCCCACACTATCTCA-3'. The amplicons were purified and sequenced with the following primers: *HBG1-seq*, 5'-TTTCCTTAGAAACCACTGCTAACTG-3'; *HBG2-seq*, 5'-CTTATTTGGAAACCAATGCTTACTA-3'; and *HBG1/2-seq*, 5'-GACGTTCCAGAAGCGAGTGT-3'. The base editing level was quantified based on Sanger sequencing (Eurofins Genomics) results by using EditR1.0.10.

For NGS, the HBG1/2 target site was amplified using the following primers: HBG-NGS forward, 5'-ACACTCTTTCCCTACACGACGCTCTTCCGATCTGACGTTCCAGAAGCGAGTGT-3', reverse, 5'-GACTGGAGTTCAGACGTGTGCTCTTCCGATCTCCCTTCCCCACACTATCTCA-3' (underlined sequences are adaptors for library construction). After cleaning-up using AMPure XP Beads (Beckman Coulter, Indianapolis, IN), the amplicons were submitted for next-generation sequencing with Amplicon-EZ by Genewiz (South Plainfield, NJ). At least 200,000 reads per amplicon were acquired to probe the types of mutations. Data were aligned to the *HBG1/2* reference sequence and analyzed using the CRISPResso2(2), a python-based genome editing analysis tool. For quantification of indel frequencies, data were aligned to the *HBG1/2* reference sequence using the Cas-Analyzer online tool, a JavaScript-based implementation for NGS data analysis. A quantification window of 140 bp around the predicted nicking site was used by setting the comparison range as 70 bp.

Flow cytometry: Cells were resuspended at $1x10^6$ cells/100 µL in FACS buffer (PBS, 1%FBS) and incubated with FcR blocking reagent (Miltenyi Biotech, Auburn CA) for ten minutes on ice. Next the staining antibody solution was added in 100 µL per 10^6 cells and incubated on ice for 30 minutes in the dark. After incubation, cells were washed once in FACS buffer. For secondary staining, the staining step was repeated with a secondary staining solution. After the wash, cells were resuspended in FACS buffer and analyzed using a LSRII flow cytometer (BD Biosciences, San Jose, CA). Debris was excluded using a forward scatter-area and sideward scatter-area gate. Single cells were then gated using a forward scatter-height and forward scatter-width gate. Flow cytometry data were then analyzed using FlowJo (version 10.0.8, FlowJo, LLC). For analysis of LSK cells, cells were stained with biotin-conjugated lineage detection cocktail (catalog # 130-092-613) (Miltenyi Biotec, San Diego, CA), antibodies against c-Kit (clone 2B8, catalog # 12-1171-83) and Sca-1 (clone D7, catalog # 25-5981-82), followed by secondary staining with APC-conjugated streptavidin (catalog # 17-4317-82) (eBioscience, San Diego, CA). Other antibodies from eBioscience included anti-mouse CD3-APC (clone 17A2) (catalog # 17-0032-82), anti-mouse CD19-PE-Cyanine7 (clone eBio1D3) (catalog # 25-0193-82), anti-mouse Ly-6G/Ly-6C (Gr-1)-PE (clone RB6-8C5) (catalog # 12-5931-82, and anti-human CD235-FITC (clone HIR2) (catalog # 11-9987-82). Anti-mouse TER-119-APC (clone TER-119) (catalog # 116211) was from Biolegend (San Diego, CA). Anti-human CD45-APC (clone 5B1) was from Miltenyi Biotec (Catalog # 130-108-020). Anti-human CD34-PE (clone 563) was from STEMCELL Technologies. Anti-human CD90-Brilliant Violet 605 (clone 5E10) (catalog # 562685) and anti-human CD38-PerCP/Cy5.5 (clone HIT2) (catalog # 561106) was from BD Biosciences.

For intracellular flow cytometry detecting human γ-globin expression, the FIX & PERM™ cell permeabilization kit (Thermo Fisher Scientific) was used and the manufacture's protocol was followed. ~5×10^6 cells or 5µL blood was stained with 0.6µg anti-human γ-globin antibody (Clone 51-7, catalog# sc-21756-PE) (Santa Cruz Biotechnology, Dallas, TX). Mouse RBCs were stained with anti-TER-119-APC antibody prior to γ-globin staining.

Globin HPLC: Lysates prepared from mouse RBCs or differentiated $CD34^+$ cells were used. Individual globin chain levels were quantified on a Shimadzu Prominence instrument with an SPD-10AV diode array detector and an LC-10AT binary pump (Shimadzu, Kyoto, Japan). Vydac 214TP™ C4 Reversed-Phase columns for polypeptides (214TP54 Column, C4 ,300 Å, 5 µm, 4.6 mm i.d. x 250 mm) (Hichrom, UK) were used. A 40%-60% gradient mixture of 0.1% trifluoroacetic acid in water/acetonitrile was applied at a rate of 1 mL/min.

Analysis of Reactive Oxygen Species (ROS) levels: Intracellular ROS levels from erythroid precursors were determined using the General Oxidative Stress Indicator CM-H2DCFDA (#C6827, LifeTechnologies), according to the manufacturer's instructions. Briefly, thalassemic $CD34^+$ cells were incubated with 10µM CM-H2DCFDA in PBS at 37°C for 1 hour and washed twice with PBS, before analysis. Oxidation of the probe can be detected by the increase of fluorescence (GFP) by flow cytometry.

Cytospin slide preparation: Cytospins of $0.3\text{-}1.0x10^5$ cells were prepared by cytocentrifugation (ROTOFIX 32, Hettich Zentrifugen) at 500 rpm for 5 minutes. Cytospins were air dried and then stained with Giemsa/May-Grünwald (Merck, Darmstadt, Germany) for 8 and 3 minutes, respectively, or subjected to imaging analysis.

HbF immunofluorescence: Slides with RBC cytospins were immersed in ice-cold methanol for 18 mins, rinsed with PBS and H_2O for 2 min each and air-dried. Slides were the incubated with anti-HbF-FITC (Santa Cruz, SC-21756) at a concentration of 20-60 µg/mL in PBS for 90 mins in humidified chambers at 37°C. Slides were then intensively rinsed with PBS and mounting Vectashield. Photos were taken with an EVOS M5000 fluorescent microscope (Invitrogen).

Real-time reverse transcription PCR: Total RNA was extracted from 5×10^6 differentiated cells or 100µL blood by using TRIzol™ reagent (Thermo Fisher Scientific) followed by phenol-chloroform extraction. QuantiTect reverse transcription kit (Qiagen) and power SYBR™ green PCR master mix (Thermo Fisher Scientific) were used. Real time quantitative PCR was performed on a StepOnePlus real-time PCR system (AB Applied Biosystems). The following primer pairs were used: mouse *RPL10* (house-keeping) forward, 5'-TGAAGACATGGTTGCTGAGAAG-3', reverse, 5'-GAACGATTTGGTAGGGTATAGGAG-3'; human γ-globin forward, 5'-GTGGAAGATGCTGGAGGAGAAA-3', reverse, 5'-TGCCATGTGCCTTGACTTTG-3'; human β-globin forward, 5'-CTCATGGCAAGAAAGTGCTCG-3', reverse, 5'-AATTCTTTGCCAAAGTGATGGG-3'; mouse β-major globin forward, 5'- ATGCCAAAGTGAAGGCCCAT-3', reverse, 5'- CCCAGCACAATCACGATCAT-3', mouse α globin forward, 5'- CTGGGGAAGACAAAAGCAAC -3', reverse, 5'- GCCGTGGCTTACATCAAAGT -3.

Measurement of vector copy number: For absolute quantification of adenoviral genome copies per cell, genomic DNA was isolated from cells using PureLink Genomic DNA Mini Kit per provided protocol (Life Technologies), and used as template for qPCR performed using the power SYBR™ green PCR master mix (Thermo Fisher Scientific). The following primer pairs were used: mgmt forward, 5'-GCTGTCTGGTTGTGAGCAGGGTCT-3', reverse, 5'-CGGGCTGGTGGAAATAGGCATTC-3'.

Off-target analyses: Circularization for *In vitro* Reporting of Cleavage Effects by sequencing (CIRCLE-Seq) was performed and analyzed as described previously(3). Data was processed using the CIRCLE-seq analysis pipeline and aligned to the mouse genome (mm10). Twenty off-target sites with highest reads numbers nominated by CIRCLE-seq were selected based on criteria described earlier (3) and subjected to further analysis using amplicon deep sequencing. Sequencing data were analyzed using the CRISPResso2 and Cas-Analyzer online tools to extract off-target base editing and indels frequencies as described above. In addition to CIRCLE-Seq, an *in silico* algorithm Cas-OFFinder (http://www.rgenome.net/cas-offinder/) (Ref PMID: 24463181) was also used for predicting off-target sites in mouse and human genomes. Five top-ranked sites were selected from each list for further amplicon deep sequencing and analyzed as described above. Primers used were listed in Supplementary Table S1.

RNA-seq analysis was performed by Omega Bioservices (Norcross, GA). Data was analyzed by Rosalind (https://rosalind.onramp.bio/), with a HyperScale architecture developed by OnRamp BioInformatics, Inc. (San Diego, CA). Reads were trimmed using cutadapt. Quality scores were assessed using FastQC. The samples were aligned to human (GRCh38.p14/hg38) and mouse (mm10) reference genome using Hisat2. Samtools used to produce bam/bai files. StringTie used for quantification and the assembly of RNA-Seq alignments into potential transcripts via annotation to gencode.v40.annotation.gff3 (for human) and gencode.vM10.annotation.gff3 (for mouse). DESeq2 was also used to calculate fold changes and adjusted *P* values (Benjamini and Hochberg adjustment method) and perform optional covariate correction. The volcano plot was generated by log-scale fold change versus adjusted p-values, and only genes meeting significance adjusted p-value < 0.01 are highlighted.

1. Wang H, Richter M, Psatha N, Li C, Kim J, Liu J, et al. A Combined In Vivo HSC Transduction/Selection Approach Results in Efficient and Stable Gene Expression in Peripheral Blood Cells in Mice. *Mol Ther Methods Clin Dev.* 2018;8:52-64.
2. Clement K, Rees H, Canver MC, Gehrke JM, Farouni R, Hsu JY, et al. CRISPResso2 provides accurate and rapid genome editing sequence analysis. *Nature biotechnology.* 2019;37(3):224-6.

3. Banskota S, Raguram A, Suh S, Du SW, Davis JR, Choi EH, et al. Engineered virus-like particles for efficient in vivo delivery of therapeutic proteins. *Cell.* 2022;185(2):250-65 e16.

Supplementary Tables (provided as Excel files)

Table S1. Potential off-target sites identified by CIRCLE-Seq. Genomic DNA from CD46/β-YAC naive mice was cleaved with recombinant SpCas9 plus sgHBG#2 RNA for CIRCLE-Seq. The top 20 sites were further analyzed by amlicon deep sequencing. Primers used for PCR and amplicon reference sequences used for analyses were listed.

Table S2. Top-scored off-target sites for sgHBG#2 guide sequence in mouse genome (GRCm38). Candidates were nominated by Cas-OFFinder and those with mismatches =< 4bp were listed. The top 5 sites highlighted in grey were amplified and further analyzed by amplicon deep sequencing. The last column shows whether those sites are overlapping with candidates nominated by CIRCLE-Seq. If yes, the corresponding ID in supplementary table 1 was indicated.

Table S3. Top-scored off-target sites for sgHBG#2 guide sequence in human genome (GRCh38). Candidates were nominated by Cas-OFFinder and those with mismatches =< 4bp were listed. The top 5 sites highlighted in grey were further analyzed by amplicon deep sequencing.

Table S4. Oligonucleotides used for cloning.

Table S5. Transcriptome analysis (human)

Table S6. Transcriptome analysis (mouse
